# Targeting G-protein-coupled receptors and gut microbiota: Ge-Lian Qi-Shen decoction elevates GLP-1 to combat non-alcoholic fatty liver disease

**DOI:** 10.1186/s13020-025-01305-9

**Published:** 2026-01-24

**Authors:** Menglei Ding, Zihan Xiao, Xionglin Hou, Zichen Luo, Zepeng Zhang, Manman Guo, Cheng Xu, Ruimin Xu, Jinjun Shan, Huiping Peng

**Affiliations:** 1 Kunshan TCM Hospital Affiliated to Nanjing University of Chinese Medicine, Kunshan, 215300 Jiangsu China; 2 Jiangsu Key Laboratory of Children’s Health and Chinese Medicine, Nanjing University of Chinese Medicine, Nanjing, 210023 Jiangsu China; 3 Suzhou Key Laboratory of Integrated Traditional Chinese and Western Medicine of Digestive Diseases, Suzhou, 215002 Jiangsu China

**Keywords:** NAFLD, GLP-1, Bitter taste receptor, TGR5, Alkaloid, Triterpene, Anthraquinone, Short chain fatty acid

## Abstract

**Background:**

Non-alcoholic fatty liver disease (NAFLD), often accompanied by insulin resistance, obesity, and hyperlipidemia, is a challenging metabolic disorder to treat. Ge-Lian Qi-Shen Decoction, a traditional Chinese herbal formula, has been clinically used to alleviate symptoms associated with NAFLD, but its underlying mechanisms remain unclear.

**Methods:**

A NAFLD model was established in C57BL/6J mice using a high-fat diet (HFD). The effects of 4-week GQD intervention at different doses on NAFLD-related symptoms were assessed using biochemical analyses, pathological sections, and oral glucose tolerance tests. ELISA and qPCR were employed to investigate the impact of GQD on serum GLP-1 levels and intestinal *Gcg* gene expression in NAFLD mice. The direct stimulatory effects of GQD on GLP-1 secretion were examined in NCI-H716 cells and HFD-fed mice. UPLC-MS/MS was used to analyze the composition of ileal contents in GQD-treated mice, and the regulatory effects of 24 identified compounds on GLP-1 secretion were evaluated. Additionally, 16S rDNA sequencing, metabolomics and fecal microbiota transplantation were utilized to explore the role of gut microbiota in GQD’s anti-NAFLD effect.

**Results:**

GQD improved HFD-induced hepatic steatosis, impaired glucose tolerance, and elevated blood lipid levels in a dose-dependent manner. It increased serum GLP-1 levels, reduced energy intake, and enhanced glucose tolerance in mice. A single dose of GQD directly elevated serum GLP-1 levels in HFD-fed mice and improved glucose tolerance in a GLP-1-dependent manner. In NCI-H716 cells, GQD promoted intracellular calcium influx and GLP-1 release by activating two G-protein-coupled receptors (GPCRs): bitter taste receptors and TGR5. Compounds such as berberine, coptisine, nuciferine, liensinine, higenamine, aurantio-obtusin, and obtusifolin in GQD activated bitter taste receptors, while maslinic acid and cycloastragenol activated TGR5, facilitating GLP-1 secretion. Furthermore, GQD gavage increased the levels of *Muribaculaceae* and *Akkermansia* in mouse feces, leading to elevated concentrations of short-chain fatty acids (SCFAs) such as acetate, propionate, butyrate, and valerate. These SCFAs potentially activated fatty acid-related GPCRs, such as GPR41, in the colon, thereby enhancing colonic *Gcg* expression. FMT experiment showed that gut microbiota can partially mediate the effect of GQD in increasing GLP-1 levels thus alleviating NAFLD.

**Conclusion:**

Some alkaloids, anthraquinones, and triterpenoids in GQD can activate GPCRs, including bitter taste receptors and TGR5, in intestinal endocrine cells, promoting GLP-1 secretion. Simultaneously, GQD regulates gut microbiota composition and metabolism, increasing SCFA levels and *Gcg* gene expression, leading to sustained elevation of GLP-1 levels. These combined effects contribute to the alleviation of NAFLD symptoms.

**Supplementary Information:**

The online version contains supplementary material available at 10.1186/s13020-025-01305-9.

## Introduction

Non-alcoholic fatty liver disease (NAFLD) is characterized by the accumulation of fat in the liver, in the absence of excessive alcohol consumption and other causes of hepatic steatosis. As obesity rates have risen globally, NAFLD has emerged as the most common chronic liver disorder worldwide, affecting 25–30% of adults [1]. Extensive clinical research has revealed strong epidemiological links between NAFLD and type 2 diabetes, with steatosis being strongly associated with insulin resistance (IR) in the liver [2]. The presence of IR can accelerate the progression of liver fibrosis, leading to cirrhosis, hepatocellular carcinoma (HCC), and death, thereby increasing the complexity and difficulty of treating NAFLD [3].

Ge-Lian Qi-Shen Decoction (GQD) is an empirically derived prescription modified from the lipid-lowering granules "Jiang Zhi Mai An" (JZMA), which was developed by the Affiliated Kunshan Hospital of Nanjing University of Chinese Medicine. JZMA has been clinically proven to be effective in the treatment of NAFLD over many years [4–6]. Itis composed of a blend of herbal ingredients (Table 1), including Astragalus membranaceus (Huangqi), Salvia miltiorrhiza (Danshen), Crataegus pinnatifida (Shanzha), Hordeum vulgare (Maiya), Alisma orientale (Zexie), Cassia obtusifolia (Juemingzi), and Nelumbo nucifera (Heye). Given the presence of IR in NAFLD patients, two additional Chinese herbs, Pueraria lobata (Gegen) and Coptis chinensis (Huanglian), were added to alleviate the disturbed glucose metabolism in NAFLD patients. Numerous studies have shown that both Gegen and Huanglian can alleviate insulin resistance by reducing inflammation, glucose metabolism and steatosis [7, 8]. And the remaining herbs in GQD also exhibit pharmacological activities, including antioxidant, anti-inflammatory and immunomodulatory properties. Many reports [9–11] have demonstrated that these herbs can improve glucose metabolism and hepatic steatosis in NAFLD and reduce liver damage.

**Table 1:** Composition of GQD

Chinese name	Plant name	Part used	Dosage (g)
Huang qi (HQ)	*Astragalus membranaceus* (Fish.) Bge. Var. mongholicus (Bge.) Hsiao	Root	15
Dan shen (DS)	*Salvia miltiorrhiza* Bge	Root and rhizome	10
Ge gen (GG)	*Pueraria lobata* (Willd.) Ohwi	Root	15
Huang lian (HL)	*Coptis chinensis* Franch	Rhizome	5
Ze xie (ZX)	*Alisma orientale* (Sam.) Juzep	Rhizome	10
Jue mingzi (JM)	*Cassia obtusifolia* L	Seed	10
Shan zha (SZ)	*Crataegus pinnatifida* Bge	Fruit	10
Mai ya (MY)	*Hordeum vulgare* L	Fruit	15
He ye (HY)	*Nelumbo nucifera* Gaertn	Leaf	10

In clinical practice, GQD has shown its unique efficacy and broad application value. However, the material basis of the formulation and the mechanism of action for reducing insulin resistance in NAFLD patients remains to be explored. Therefore, this study explores the formula from multiple perspectives, aiming to uncover the mechanisms of action through which the GQD formula treats NAFLD.

## Materials and methods

### Materials

HQ(20220802), HL(20211105), JMZ(20221223) were all purchased from Tongling Hetian Traditional Chinese Medicine Co. in Anhui, China. DS(20220701-01), GG(20211101-01) were both purchased from Guizhou Tongde Pharmaceutical Co. in Guizhou, China. ZX(221031), SZ(230809), MY(270404), HY(220628) were all purchased from Suzhou Tianling Chinese Herbal Medicine Co. Ltd. in Suzhou, China. Triamterene (HY-B0575), liensinine (HY-N0484), lactisole (HY-W030796A), probenecid (HY-B0545) and phenylthiocarbamide (HY-Y0351) were purchased from MedExpress. Salvianic acid A (B20260), protocatechualdehyde (B21613), caffeic acid (B20660), puerarin (B20446), daidzin (B20226), calycosin-7-glucoside (B20847), rosmarinic acid (B20862), jatrorrhizine (B75604), coptisine chloride (B21438), salvianolic acid B (B20261), berberrubine (B21377), daidzein (B20227), nuciferine (B20500), palmatine hydrochloride (B21433), salvianolic acid A (B20260), berberine hydrochloride (B21449), luteolin (B20888), quercetin (B20527), kaempferol (B21126), isorhamnetin (B21554), astragaloside IV (B20564), chrysophanol (B20238), formononetin (S31511), astragaloside II (B20566), aurantio-obtusin (B20180), rhein (B20245), astragaloside I (B20565), obtusin (B20658), obtusifolin (B20858), 23-acetyl alisol C (B21764), emodin (B20240), cycloastragenol (B20548), cryptotanshinone (B21586), maslinic acid (B21135), tanshinone IIA (B20257), ursolic acid (B21403) and alisol B 23-acetate (B21641) were purchased from Shanghai yuanye Bio-Technology Co., Ltd.

### Preparation of GQD

The nine herbs listed in Table 1 were soaked in water at a ratio of 1:10 (w/v) for 1 h, then refluxed and extracted twice, each for 1 h. The resulting decoctions were combined and concentrated to achieve a final crude drug concentration of 2 g/mL.

### Animal care and study

All animal experiments were conducted following approval from the Institutional Animal Care and Use Committee of Nanjing University of Chinese Medicine (permit NO. 202307A059) and in accordance with the guidelines outlined by the National Institutes of Health for the use of experimental animals. 8-week-old male C57BL/6J mice were procured from Beijing Vital River Laboratory Animal Technology Co. Ltd. They were housed in a specific pathogen-free facility with individual ventilated cages.

The dosing regimens for mice were calculated from human clinical doses using the body surface area normalization method. The human-equivalent clinical dose of GQD translates to 13 g/kg per day in mice. In this experiment, three different gavage doses were set: 20 g/kg (GQD_H), 10 g/kg (GQD_M), and 5 g/kg (GQD_L). These three dose levels encompass the clinically equivalent dose [12]. Initially, the mice underwent a 3-day acclimation period on a standard chow diet (SWS9102, Jiangsu Xietong Pharmaceutical Bioengineering CO., LTD., Nanjing, China). Subsequently, they were either continued on the chow diet or switched to a high-fat diet (HFD, D12492, Research Diets Inc., New Brunswick, NJ, USA). After 8 weeks, the mice were allocated into six groups and treated as follows: (1) Chow group: maintained on a chow diet with daily gavage of 0.2 mL sterile water; (2) HFD group: maintained on an HFD with daily gavage of 0.2 mL sterile water; (3) HFD + GQD_H: maintained on an HFD with daily gavage of GQD_H; (4) HFD + GQD_M: maintained on an HFD with daily gavage of GQD_M; (5) HFD + GQD_L: maintained on an HFD with daily gavage of GQD_L; (6) HFD + MET: maintained on an HFD with daily gavage of 200 mg/kg metformin. The treatment period lasted for 4 weeks. The body weight of the mice was recorded weekly, while their daily food intake was carefully monitored. The weight gain was calculated by subtracting the initial weight from the weight measured at each time point. For mice on a high-fat diet (HFD), their daily caloric intake was determined by multiplying the daily food intake per mouse (in grams) by the energy density of the diet, which is 5.24 kcal/g.

For the 8-week treatment study, NAFLD mice were generated by feeding an HFD for 8 weeks. After the initial 8-week induction, NAFLD mice were randomly assigned to receive daily gavage of sterile water on an HFD (HFD), or GQD_H, GQD_M, or GQD_L on an HFD for an additional 8 weeks. The chow group remained on a chow diet throughout the entire study period and received a daily gavage of 0.2 mL sterile water during the final 8 weeks.

### Oral glucose tolerance test (OGTT) and GLP-1 measurement for mice

OGTT was conducted following established protocols during the 11th week of the study [13].

The measurement of serum GLP-1 in pharmacological efficacy study and fecal microbiota transplantation study was also referenced from previous studies [14]. Mice were fasted for six hours, then were administered 3 mg/kg of sitagliptin (a DPP-4 inhibitor) by gavage. After one hour, they were orally administered 2 g/kg of glucose, and blood samples were collected from the retro-orbital venous plexus 30 min later. Serum was obtained after centrifugation, and GLP-1 concentration was measured using an ELISA assay kit.

### Histopathology and biochemical analyses

Liver and intestinal histopathological staining, liver lipid contents (TG and TC), and serum aminotransferase levels (ALT and AST) were assessed according to established methodologies described in previous reports [15].

### Intestinal mRNA extraction and real-time quantitative PCR (qPCR)

The extraction, reverse transcription, and real-time qPCR methods for mRNA in the ileum and colon were referenced from the previous study [15]. The primer sequences are provided in Table S1 of the Supplementary Information.

### Acute effects of GQD on GLP-1 levels and glucose tolerance

HFD-fed mice (35 ± 3 g) were fasted for six hours and orally administered sitagliptin (3 mg/kg). Thirty minutes later, they received GQD_H, GQD_M, GQD_L, or sterile water by gavage. After an additional 30 min, blood samples were collected from the orbital venous plexus (0-min time point). The mice were then given an oral glucose load (2 g/kg), and further blood samples were collected at 15 and 30 min. GLP-1 levels were measured according to the manufacturer’s instructions.

To examine whether the acute stimulatory effect of GQD_H is mediated by bitter receptors and TGR5, HFD-fed mice (35 ± 3 g) were fasted for six hours and orally administered sitagliptin (3 mg/kg). Thirty minutes later, the mice received vehicle, GQD_H, GQD_H with 50 mg/kg probenecid, or GQD_H with 50 mg/kg triamterene by oral gavage. After an additional 30 min, blood samples were collected from the orbital venous plexus (0-min time point). The mice were then given an oral glucose load (2 g/kg), and further blood samples were collected at 15 and 30 min. GLP-1 levels were measured according to the manufacturer’s instructions.

To assess the acute effect of GQD on glucose tolerance, HFD-fed mice (35 ± 3 g) were fasted overnight and administered GQD_H, GQD_M, GQD_L, or sterile water by oral gavage. Thirty minutes later, blood glucose at the 0-min time point was measured from the tail tip, after which the mice were given an oral glucose load (2 g/kg). Blood glucose levels were subsequently measured at 15, 30, 60, and 120 min.

For GLP1R inhibition experiment, HFD-fed mice weighing (35 ± 3 g) were fasted overnight and 25 nmol/kg Exendin (9–39) amide (HY-P0264, MedChemExpress) or normal saline were injected intraperitoneally [16]. Five minutes later, the mice were administered GQD_H or sterile water by oral gavage. After an additional 30 min, blood glucose at the 0-min time point was measured from the tail tip, followed by an oral glucose load (2 g/kg). Blood glucose levels were subsequently measured at 15, 30, 60, and 120 min.

### Cell culture and viability assay

Human NCI-H716 cells (ATCC-CCL-251, the American Type Culture Collection, Manassas, USA) were cultured in suspension in RMPI 1640 medium supplemented to contain 10% FBS. The cells were maintained at a density of approximately 500,000 cells/mL and were passaged every 3 days.

Cell viability was assessed using the CCK8 assay. Initially, a 96-well plate was coated with incomplete RMPI 1640 medium containing 0.32 mg/mL Matrigel, with 50 μL added to each well. Following incubation at 37 °C for 1 h, the medium was aspirated, and the wells were rinsed with PBS. Subsequently, approximately 8,000 NCI-H716 cells were seeded into each well using complete RMPI 1640 medium. After overnight adherence of the cells, the medium was replaced with incomplete medium containing varying concentrations of drugs, and the CCK8 assay was conducted following the manufacturer’s instructions.

### GLP-1 secretion and intracellular calcium measurement in NCI-H716 cells

For the GLP-1 secretion experiment, each well of a 24-well plate was added with 250 μL of incomplete RMPI 1640 medium containing 0.32 mg/mL Matrigel. After incubating at 37 °C for 1 h and removing the medium, 100,000 NCI-H716 cells were seeded into each well. Before treatment, the cells were serum-starved overnight. The drugs were either prepared in Krebs–Ringer HEPES (KRH) buffer or dissolved in DMSO and then diluted in KRH buffer. Following a 60-min treatment with drug-containing or drug-free KRH buffer, cell supernatants were collected, and GLP-1 concentrations were determined following the manufacturer’s instructions. Additionally, cells were collected to measure protein levels for calibration of inter-well cell quantity differences. The mean ratio of GLP-1 concentration to protein level in control wells was used to normalize all wells, thereby calculating the fold change in GLP-1 secretion relative to the control group for different treatment groups.

For intracellular calcium measurement, NCI-H716 cells were seeded into a 96-well black plate pre-coated with Matrigel. The cells were serum-starved overnight and treated with drugs dissolved in KRH buffer for 60 min. Intracellular calcium levels were measured according to the manufacturer’s instructions. The relative fluorescence units (RFU) for each well were obtained by normalizing to the mean fluorescence intensity of the control wells.

### Small interfering RNA (siRNA) infection in NCI-H716 cells

Three siRNA oligonucleotides were designed for each of the *TAS2R38* and *GPBAR1* genes (Table S2). The siRNA with the highest knockdown efficiency, as determined by qPCR analysis (Fig. S1), was selected for subsequent experiments. For siRNA transfection, NCI-H716 cells were seeded into a 96-well or 24-well plate pre-coated with Matrigel. According to the manufacturer's instructions for Lipofectamine^™^ 3000 (Thermo Fisher Scientific Inc., Waltham, MA, USA), 3 pmol of siRNA was added to each well of the 96-well plate, and 15 pmol was added to each well of the 24-well plate. After 48 h of transfection, the medium was replaced with either drug-containing or drug-free KRH buffer for subsequent calcium ion or GLP-1 concentration assays.

### Dual-luciferase reporter assay

HEK293T cells were cultured in high-glucose DMEM supplemented with 10% fetal bovine serum and seeded into 96-well plates at 40,000 cells/well. Cells were transfected using Lipofectamine^™^ 3000 with the following plasmids per well: 20 ng pGL4.29[luc2P CRE Hygro] vector (primary reporter gene, Promega), 20 ng pcDNA3.1-*GPBAR1* (human TGR5 overexpression), and 10 ng pRL-TK (Renilla luciferase internal control, Promega). After 6 h incubation, the medium was replaced with high-glucose DMEM containing test compounds. Following 24 h treatment, dual-luciferase activity was measured using the Dual-Luciferase^®^ Reporter Assay System (Promega).

### Composition analysis of GQD and its components distribution in the ileum

For GQD composition analysis, 800 μL methanol was added into 200 μL GQD, and the mixture was vortexed for 30 s. The supernatant obtained after centrifugation at 18,000 rpm for 10 min was used for analysis. For components distribution analysis in the ileum, mice given GQD_H were sacrificed at 30, 60, 120, and 240 min, and the ileal contents were collected. 50 mg ileal contents were homogenized in 500 μL of 70% methanol solution and centrifuged at 18,000 rpm for 10 min. 400 μL supernatant was then vacuum concentrated to dryness, and the residue was re-dissolved in 80 μL of 70% methanol solution, vortexed for 5 min, centrifuged at 18,000 rpm for 10 min, and the supernatant was used for analysis.

The chemoprofile of GQD was performed by UPLC-LTQ-Orbitrap MS/MS, coupled with an HSS-T3 column (2.1 × 100 mm, 1.7 μm). The temperature of column oven was set 30 ℃, and the mobile phase was water with 0.1% formic acid (A) and acetonitrile with 0.1% formic acid (B). The elution gradient was: 0 min, 5% B, 18 min, 50% B, 23 min, 95% B, 26 min, 5% B, 30 min, 5% B, and the flow rate was 0.2 mL/min. An ESI ion source was adopted, and the parameters were set as: source voltage, 4 kV, capillary voltage, 35 V, tube lens, 100 V. The analyzer of MS1 was FTMS, and other parameters were set as: resolution, 30000, scan type, full MS, nCE, 35 eV, scan range, m/z 50–1500.

### 16S rDNA

Total DNA for sequencing was extracted using Stool DNA Kit (OMEGA Bio-Tek, Norcross, GA, USA). According to the conserved region of 16S rDNA/ITS2 sequence, the extracted microbial DNA was used as template for one-step PCR using Phusion enzyme for 35 cycles, and the sequencing universal connector and sample-specific Barcode sequence were added to the amplified product of the target region by PCR. The PCR amplified product was detected and recovered using AxyPrep PCR Cleanup Kit by electrophoresis on 1.5% agarose gel for detection, and the target fragments were recovered using the AxyPrep PCR Cleanup Kit recovery kit. The purified PCR products were used to quantify and mix the library on a Promega QuantiFluor Fluorescence Quantification System using Quant-iT PicoGreen dsDNA Assay Kit. Finally, we used Illumina Sequencing Platform 250PE to perform bipartite sequencing according to standard operations.

### Targeted metabolomics analyses

Targeted metabolomics analyses were performed by UltiMate^®^ 3000 UPLC system (DIONEX, Sunnyvale, CA, USA) coupled with a TSQ Vantage^™^ triple quadrupole mass spectrometer (Thermo Fisher Scientific Inc.).

For SCFA analysis, 500 μL 50% acetonitrile was added to 10 mg colonic contents and shaken for 5 min. The homogenate was then centrifuged for 10 min at 13,200 rpm. Afterward, 5 μL internal standard (10 μg/mL caproic acid-d3) was added into 40 μL supernatant. Next, 20 μL 200 mM 3-nitrophenylhydrazine (3NPH) solution and 20 μL of 120 mM N-(3-dimethylaminopropyl)-N’-ethylcarbodiimide (EDC) solution were added. The mixture was incubated at 40 °C for 30 min, then centrifuged at 13,200 rpm for 10 min. To the resulting supernatant (20 μL), 180 μL of 50% acetonitrile was added and vortexed thoroughly. Finally, the mixture was centrifuged at 18,000 rpm for 10 min, and 100 μL supernatant was collected for LC–MS/MS analysis. Chromatographic separation of SCFA was performed on a BDS HYPERSIL C_18_ column (2.1 × 100 mm, 2.4 μm, Thermo Scientific). Mobile phase A was water containing 0.1% formic acid (v/v), and mobile phase B was acetonitrile containing 0.1% formic acid (v/v). The mobile phase gradient was programmed as follows: 0 ~ 3 min, 10% B; 3 ~ 10 min, 10 ~ 35% B; 10 ~ 12 min, 35 ~ 95% B; 12 ~ 14 min, 95% B; 14 ~ 14.5 min, 95 ~ 10% B; 14.5 ~ 16 min, 10% B, with a flow rate of 0.3 mL/min. The column oven temperature was set to 50 °C. For the mass spectrometer detector, the parameters were set as follows: ESI (−); spray voltage, 3.5 kV; capillary temperature, 320 °C; sheath gas flow rate, 45 arb; auxiliary gas flow rate, 25 arb; and vaporizer temperature, 400 °C. SRM parameters for SCFAs are shown in Table 2.

**Table 2:** SRM parameters of targeted SCFAs and bile acids

Analytes	Parent (m/z)	Product (m/z)	S-Lens/V	CE/eV
Lithocholic acid (LCA)	375.20	375.20	149	15
Hyodeoxycholic acid (HDCA), ursodeoxycholic acid (UDCA), chenodeoxycholic acid (CDCA), deoxycholic acid (DCA)	391.20	391.20	132	15
CDCA-d4	507.20	80.00	149	64
Acetate	194.1	137.1	40	20
Propionate	208.1	165.1	50	20
Butyrate	222.1	137.1	65	20
Valerate	236.1	137.1	75	24
Caproic acid-d3	253.1	137.1	75	25

For bile acid analysis, 500 μL 50% methanol and 10 μL internal standard (CDCA-d4, 1 μg/mL) were added to 20 mg colonic contents, followed by homogenization for 5 min. The homogenate was then centrifuged for 10 min at 13,200 rpm. Next, 400 μL supernatant was transferred to a new centrifuge tube and dried at 45 °C. After drying, 80 μL 70% methanol were added, and the mixture was shaken for 5 min before centrifugation at 18,000 rpm for 10 min. Finally, 60 μL of the supernatant was collected for analysis. Chromatographic separation of bile acids was performed on an Acquity UPLC^®^ BEH C_18_ column (2.1 × 100 mm, 1.7 μm, Waters Co., Milford, MA, USA). Mobile phase A consisted of water with 0.1% formic acid (v/v), while mobile phase B consisted of acetonitrile and methanol (95:5) with 0.1% formic acid (v/v). The mobile phase gradient was programmed as follows: 0 ~ 2 min, 26% B; 2 ~ 4 min, 26 ~ 30% B; 4 ~ 5 min, 30 ~ 35% B; 5 ~ 18 min, 35 ~ 60% B; 18 ~ 21 min, 60 ~ 100% B; 21 ~ 22 min, 100 ~ 26% B; and 22 ~ 25 min, 26% B, with a flow rate of 0.4 mL/min. The column oven temperature was maintained at 45 °C. For the mass spectrometer detector, the parameters were set as follows: ESI (−); spray voltage, 3.5 kV; capillary temperature, 320 °C; sheath gas flow rate, 45 arb; auxiliary gas flow rate, 25 arb; and vaporizer temperature, 400 °C. SRM parameters for SCFAs are shown in Table 2.

### Fecal microbiota transplantation (FMT) experiment

A total of 28 C57BL/6J mice were randomly assigned to four groups: model donor group (Don_HFD, n = 6), GQD donor group (Don_GQD, n = 6), model recipient group (Rec_HFD, n = 8), and GQD recipient group (Rec_GQD, n = 8). After 7 weeks of HFD feeding, donor mice received oral gavage of 0.2 mL sterile water or GQD_H according to group assignment. Recipient mice were administered 0.2 mL of a mixed antibiotic solution (neomycin sulfate, ampicillin, and metronidazole, each at 5 mg/mL; vancomycin, 2.5 mg/mL) to deplete their original gut microbiota. One week later, donor mice continued oral gavage as before, and fresh feces were collected using sterile instruments. The feces were suspended in sterile PBS to a concentration of 200 mg/mL, homogenized, and the tubes were kept upright to allow large particles to settle naturally. The supernatant was collected and centrifuged at 5000 rpm for 5 min, the supernatant discarded. The pellet was resuspended in sterile PBS and centrifuged; this resuspension–centrifugation wash was performed twice, and the final pellet was resuspended in an equal volume of sterile PBS. The resulting suspension was administered to recipient mice via oral gavage at a dose of 0.2 mL per mouse.

### Statistical analysis

Graphpad 9.0 was used for statistical difference test of other data. For body weight gain and OGTT curves, two-way ANOVA test with Tukey's multiple comparisons test was used. For other indicators, one-way ANOVA test with Tukey's multiple comparisons test or Welch ANOVA test with Dunnett T3 multiple comparisons test were used, depending on the data homogeneity of variances.

## Results

### GQD alleviated NAFLD-related symptoms in HFD-fed mice and reduced calorie intake

**Fig. 1:**
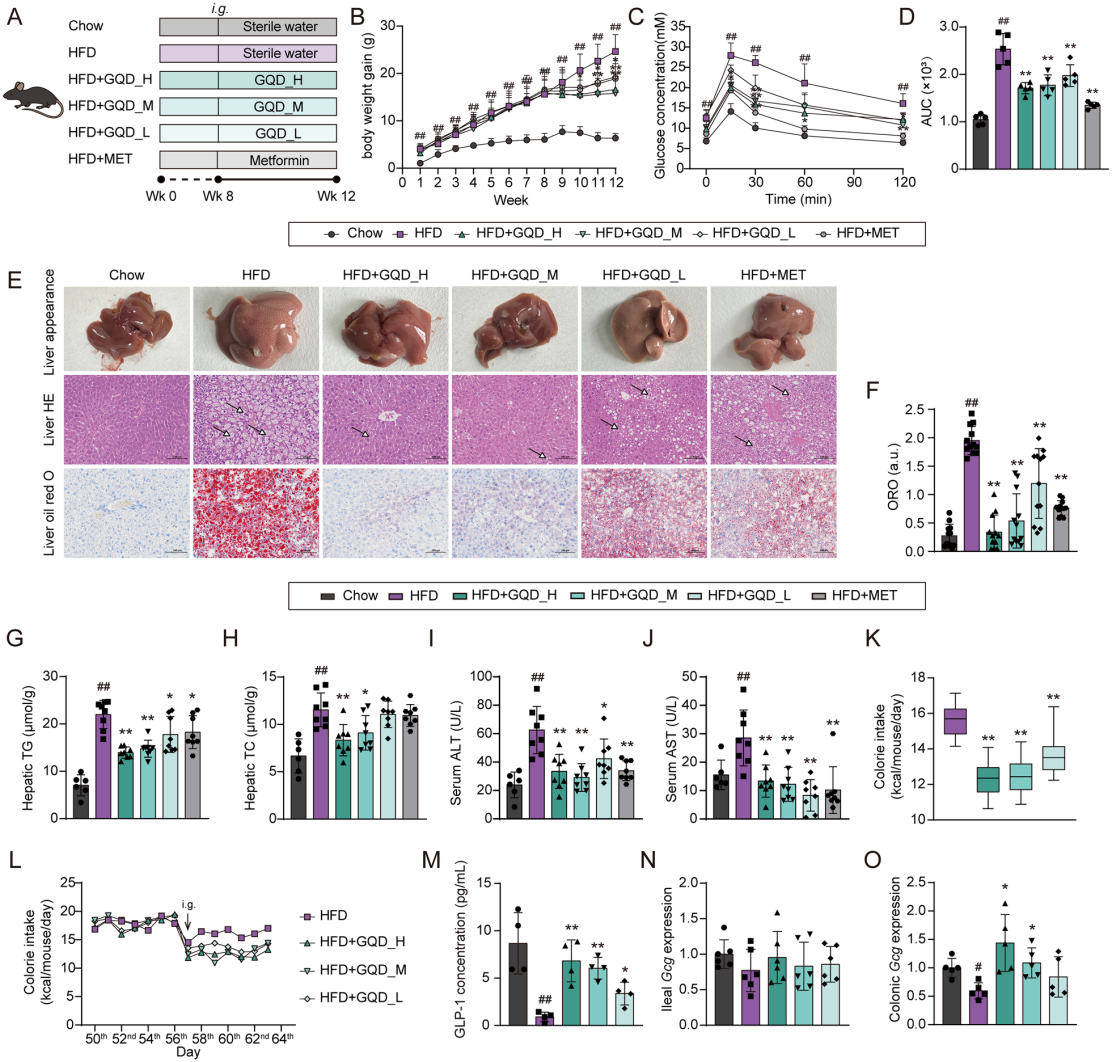
C57 BL/6 J mice were treated with Chow or HFD for 12 weeks, including 4 weeks of GQD_H, GQD_M, or GQD_L administration, respectively. **A** Animal experimental design. **B** Body weight gain (n = 6 ~ 9). **C** Blood glucose curves in OGTT (n = 5). **D** The area under the curve (AUC) of blood curves in OGTT (n = 5). **E** Representative liver appearance and liver tissue sections stained with H&E and Oil Red (200 ×, scale bars: 100 µm). Hollow arrows with solid lines represent lipid droplets. **F** Quantification of neutral lipids by oil red O in liver (n = 12). **E** Hepatic contents of TG and TC (n = 6 ~ 8). **F** Serum concentrations of ALT and AST (n = 6 ~ 8). **G** Hepatic TG contents (n = 6 ~ 8). **H** Hepatic TC contents (n = 6 ~ 8). **I** Serum ALT levels (n = 6 ~ 8). **J** Serum AST levels (n = 6 ~ 8). **K** The change in daily calorie intake per mouse before and after gavage with GQD. **L** Daily calorie intake per mouse over the four weeks of gavage. **M** Serum GLP-1 concentration in HFD fed mice after 4 weeks of GQD intervention (n = 4). **N** *Gcg* expression in mice ileum (n = 6). **O** *Gcg* expression in mice colon (n = 5). Compared with the Chow group, #*p* < 0.05, ##*p* < 0.01. Compared with the HFD group, *p < 0.05, **p < 0.01

Male C57BL/6 J mice were fed a high-fat diet for 8 weeks and then administered different doses of GQD or metformin (Fig. 1A). As shown in Fig. 1B, compared to a chow diet, mice fed with an HFD exhibited significantly increased weight gain compared to those on a chow diet (*p* < 0.01). Following weight measurements at the 8th week, mice on the HFD received varying doses of GQD or 200 mg/kg metformin, respectively. Results revealed that starting from the 10th and 11th weeks respectively, GQD_H and GQD_M significantly reduced body weight gain in mice (*p* < 0.01 or 0.05); metformin decreased body weight gain at the 12th week (*p* < 0.05); while GQD_L showed a trend towards body weight reduction, though statistically insignificant. Furthermore, the OGTT experiment (Fig. 1C, D) indicated that GQD improved glucose tolerance in HFD-fed mice in a dose-dependent manner (*p* < 0.01). Observation of liver appearance, as well as histological examination of liver tissue using H&E and Oil Red O staining, revealed significant hepatic lipid accumulation in HFD-fed mice, mitigated by metformin and different doses of GQD (Fig. 1E, F). Quantitative analysis of hepatic lipids (Fig. 1G, H) demonstrated that metformin and all doses of GQD significantly reversed the increase in liver total triglyceride (TG) content induced by the HFD (*p* < 0.01 or 0.05), while GQD_H and GQD_M markedly reversing the elevation in liver total cholesterol (TC) levels (*p* < 0.01 or 0.05). Moreover, metformin and different doses of GQD reduced serum ALT and AST levels in HFD-fed mice, indicating alleviation of liver damage and dyslipidemia (Fig. 1I, J). During the 12-week feeding period, we also monitored the daily food intake of mice to assess changes in calorie intake. As depicted in Fig. 1K, during the week preceding gavage, the calorie intake per mouse per day was nearly consistent between the HFD and GQD groups. However, following gavage with varying doses of GQD, a reduction in daily calorie intake per mouse was observed. Illustrated in Fig. 1L, over the 4-week gavage duration, mice administered different doses of GQD displayed a significantly lower daily calorie intake compared to those in the HFD group (*p* < 0.01). Notably, the effects of GQD_H and GQD_M were more pronounced than those of GQD_L.

### GQD improved GLP-1 levels in HFD-fed mice

**Fig. 2:**
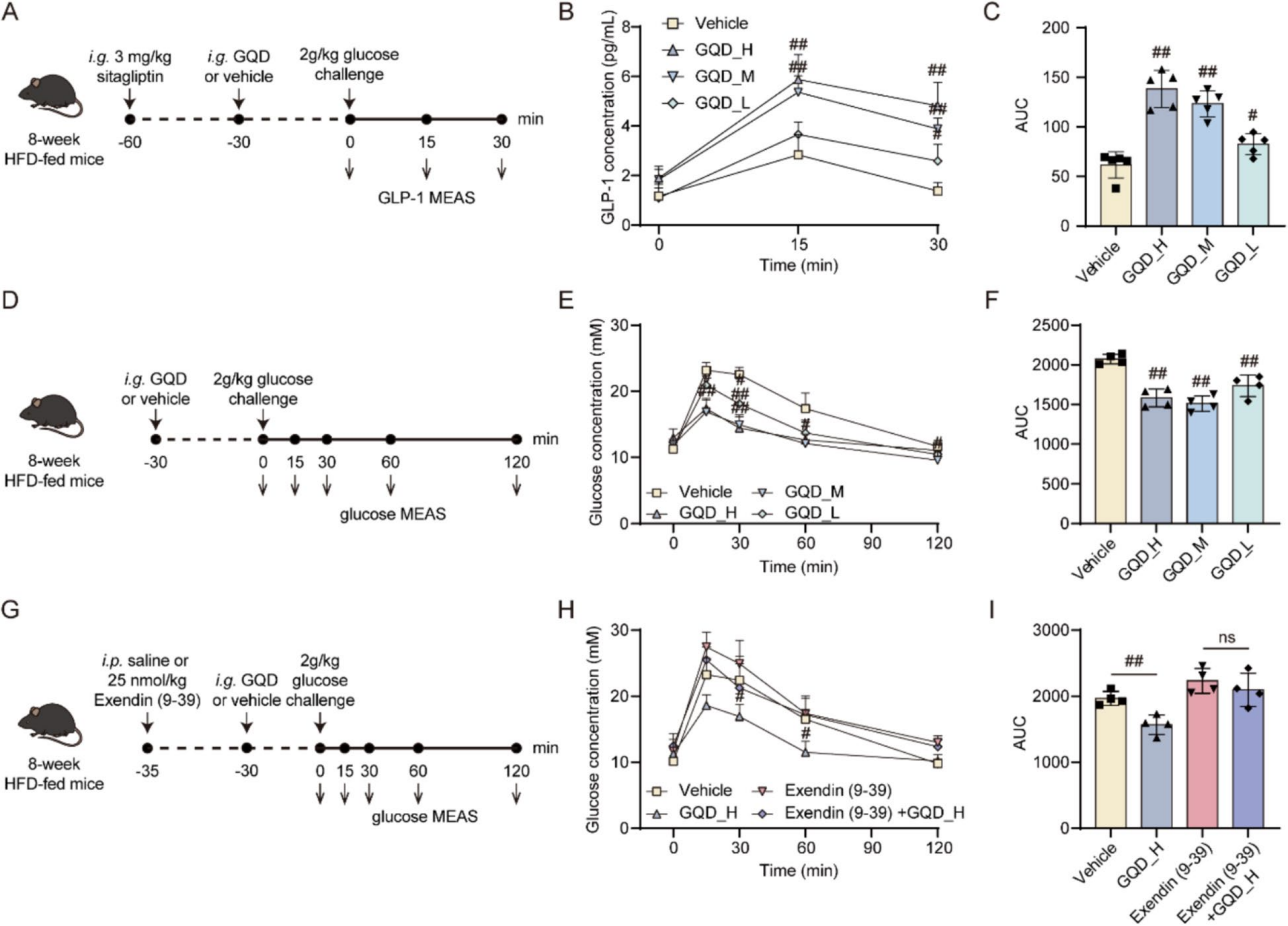
**A** Schematic of the acute experiment assessing the stimulatory effect of GQD on GLP-1 release in 8-week HFD-fed mice. **B** Time-course curve of serum GLP-1 levels (n = 5). **C** Area under the GLP-1 curve (n = 5). **D** Schematic of the acute GQD administration experiment evaluating glucose tolerance in 8-week HFD-fed mice. **E** Time-course curve of blood glucose levels (n = 4). **F** Area under the GLP-1 curve (n = 4). **G** Schematic of the experiment evaluating whether the acute effect of GQD on glucose tolerance in 8-week HFD-fed mice is dependent on GLP-1. **H** Time-course curve of blood glucose levels (n = 4). **I** Area under the GLP-1 curve (n = 4). Compared with the Vehicle group, #*p* < 0.05, ##*p* < 0.01

Given the enhanced insulin sensitivity and reduced calorie intake observed in HFD-fed mice after 4 weeks of GQD treatment, we hypothesize that GQD may stimulate GLP-1 secretion in these mice. GLP-1, produced by intestinal L cells, is known to bind GLP1R in pancreatic β cells and the hypothalamic dorsomedial nucleus, thereby regulating insulin secretion and appetite [17]. As shown in Fig. 1M, GQD induced a dose-dependent increase in GLP-1 levels in HFD-fed mice (*p* < 0.01 or 0.05). GLP-1 is produced by the processing of proglucagon (encoded by the *GCG* gene), and it has been reported that the distal gut (ileum and colon) is the primary site of GLP-1 production [18, 19]. Therefore, the mRNA expression of *Gcg* gene in mice ileum and colon was investigated. As shown in Fig. 1 N and O, both GQD_H and GQD_M significantly increased the expression of the *Gcg* gene in the colon (*p* < 0.05), while no changes were observed in the ileum. To further evaluate the durability of these effects, we performed an additional 8-week intervention in mice following 8 weeks of HFD induction (Fig. S2A). As shown in Fig. S2B–J, long-term GQD treatment consistently reduced body weight gain (*p* < 0.05 or 0.01), improved glucose tolerance (*p* < 0.05), enhanced GLP-1 secretion (*p* < 0.05 or 0.01), and alleviated hepatic steatosis (*p* < 0.05 or 0.01), accompanied by decreases in serum ALT and AST levels (*p* < 0.05 or 0.01). These findings indicate that the metabolic benefits of GQD are sustained during extended treatment. In addition, the acute effects of GQD on GLP-1 secretion was assessed. As shown in Fig. 2A, HFD-fed mice received an acute administration of GQD_H, GQD_M, or GQD_L, and the results (Fig. 2B and C) demonstrated that GQD directly stimulated GLP-1 secretion in a dose-dependent manner (*p* < 0.01 or 0.05). Moreover, acute administration of GQD prior to glucose loading improved glucose tolerance in a dose-dependent manner (*p* < 0.01, Fig. 2D–F). Finally, pre-treatment with Exendin (9–39) amide, a GLP1R inhibitor, reversed the glucose tolerance-enhancing effect of acute GQD_H treatment, indicating that the effect is GLP-1 dependent (Fig. 2G–I).

### GQD stimulated GLP-1 secretion through activation of bitter taste receptor and TGR5

**Fig. 3:**
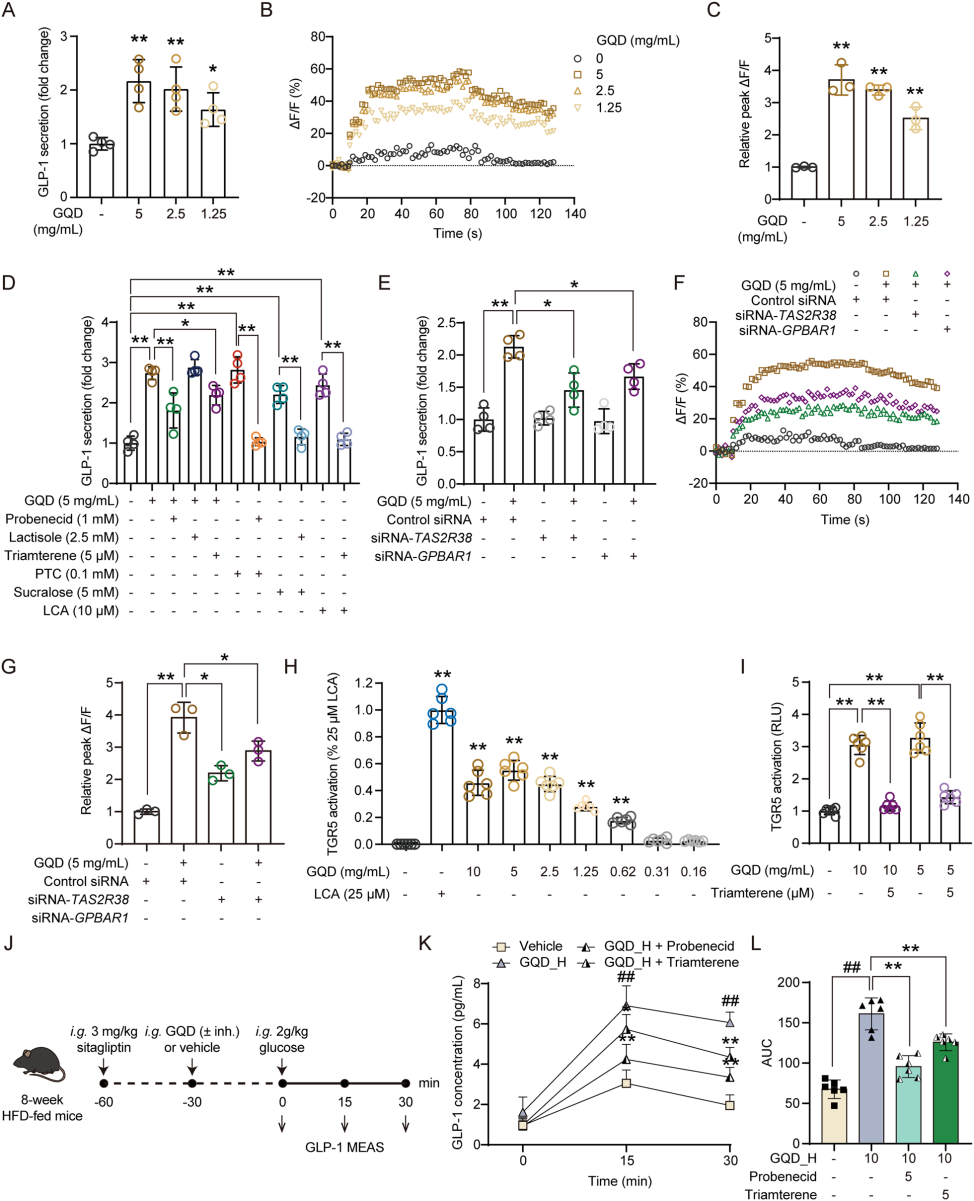
**A** The stimulation of GLP-1 release in NCI-H716 cells by GQD (n = 4). **B** The promotion of Ca^2^⁺ influx in NCI-H716 cells by GQD (n = 4). **C** Relative peak ΔF/F representing the maximal fluorescence change during Ca^2^⁺ influx (n = 3). **D** The effect of inhibitors on GQD-stimulated GLP-1 secretion in NCI-H716 cells (n = 4). **E** The effect of siRNA on GQD-stimulated GLP-1 secretion in NCI-H716 cells (n = 4). **F** The effect of siRNA on GQD-promoted calcium influx in NCI-H716 cells (n = 3). **G** Relative peak ΔF/F representing the maximal fluorescence change during Ca^2^⁺ influx (n = 3). **H** Assessment of the direct agonistic effect of GQD on TGR5 in HEK 293 T cells (n = 6). **I** Assessment of the reversal of GQD-induced TGR5 activation by Triamterene in HEK 293 T cells. **J** Schematic of the experiment evaluating the attenuation of GQD-induced GLP-1 secretion in HFD-fed mice by probenecid (50 mg/kg) and triamterene (50 mg/kg). **K** Time-course curve of serum GLP-1 levels (n = 6). **L** Area under the GLP-1 curve (n = 6). Compared between the two groups or with the GQD_H group, *p < 0.05, **p < 0.01. Compared with the Vehicle group, ##*p* < 0.05

According to reports, the components of traditional Chinese medicine may stimulate calcium influx in intestinal endocrine cells through various GPCRs, thus promote GLP-1 secretion [20–25]. To investigate the regulation of GLP-1 secretion from human intestinal L cells by GQD and explore its mechanisms, we treated NCI-H716 cells with different concentrations of GQD. The results of cell viability assays (Fig. S3A) showed that treatment with GQD at concentrations of 5 mg/mL did not affect the viability of NCI-H716 cells. As shown in Fig. 3A–C, treatment with 5, 2.5, and 1.25 mg/mL of GQD for 60 min significantly promoted GLP-1 secretion and calcium influx in NCI-H716 cells (*p* < 0.01 or 0.05). As shown in Fig. 3D, the bitter receptor inhibitor probenecid, the sweet receptor inhibitor lactisole, and the TGR5 inhibitor triamterene respectively reversed the increase in intracellular calcium levels and GLP-1 release stimulated by phenylthiocarbamide (PTC), sucralose, or lithocholic acid (LCA) in NCI-H716 cells (p < 0.01). Both probenecid and triamterene significantly attenuated the GQD-induced increase in GLP-1 release (*p* < 0.01 or 0.05), whereas lactisole showed no such effect. As shown in Fig. 3E–G, using siRNA to interfere with the expression of the *TAS2R38* gene or *GPBAR1* gene (which encodes the TGR5 protein) also significantly reduced the increase in intracellular calcium levels and GLP-1 secretion stimulated by GQD (*p* < 0.01). We further established a dual-luciferase reporter assay in HEK 293 T cells based on the TGR5-CRE system and found that GQD treatment significantly increased reporter activity in a concentration-dependent manner (*p* < 0.01, Fig. 3H and Fig. S3B). Moreover, triamterene reversed the effect of GQD (*p* < 0.01, Fig. 3I), indicating a direct activation of TGR5 by GQD. Finally, in the acute GLP-1 secretion assay, both probenecid and triamterene were found to attenuate the GQD-induced increase in GLP-1 secretion in HFD-fed mice (*p* < 0.01, Fig. 3J–L). These results suggest that GQD may stimulate GLP-1 secretion by activating bitter receptor and TGR5.

### Compounds analysis in GQD

**Fig. 4:**
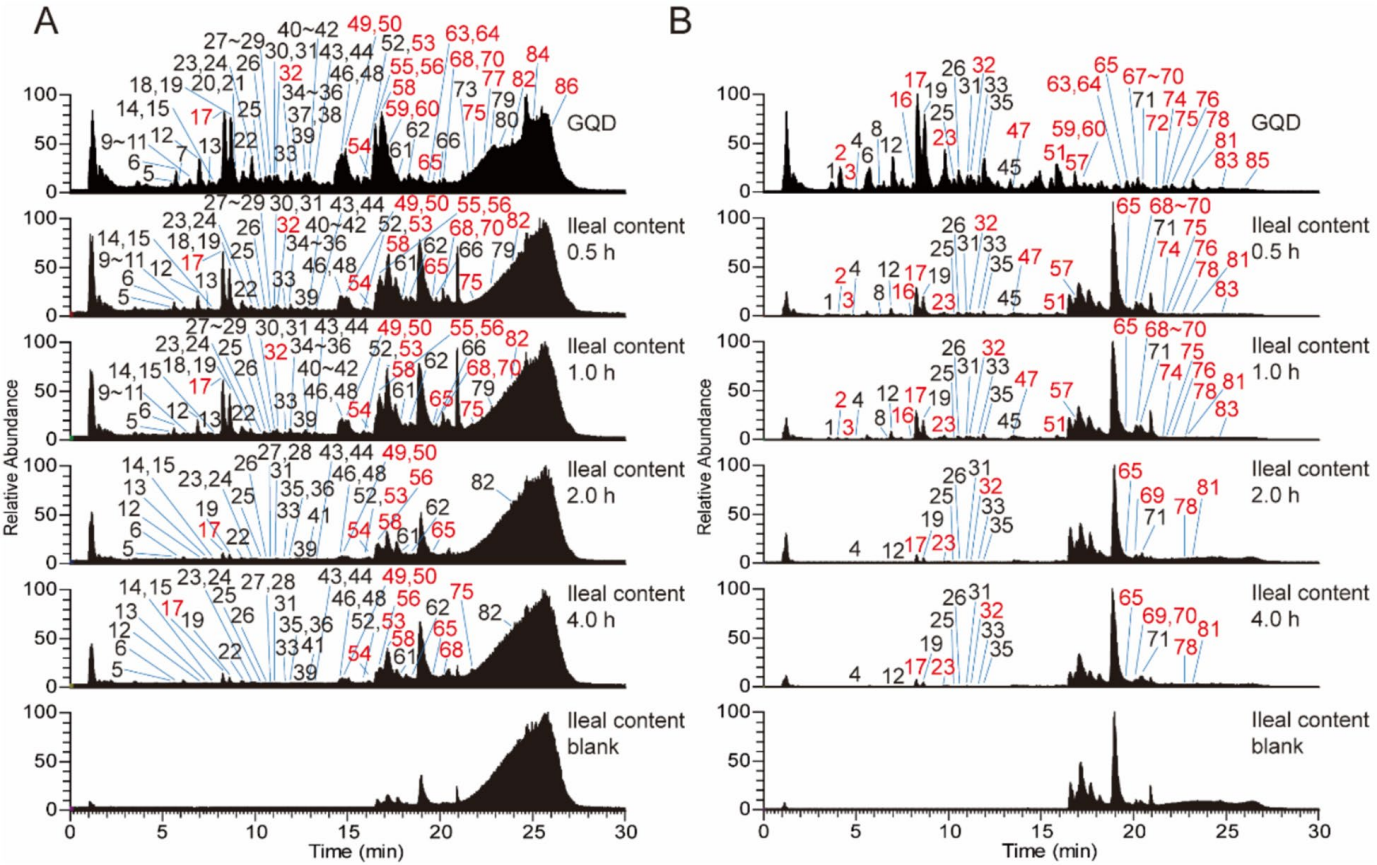
Compounds analysis in GQD. **A** The LC–MS/MS chemoprofiles of GQD and the ileum contents after GQD gavage in positive ion mode. **B** The LC–MS/MS chemoprofiles in negative ion mode

To investigate which components in GQD stimulate GLP-1 release, UPLC-MS/MS was used to analyze the chemical composition of GQD (Fig. 4A, B). A total of 86 compounds were annotated in the decoction, among which, 39 were matched with retention times using reference standards (Fig. 4, Table 3). From GQD, a total of 36 alkaloids were identified, comprising 5 aporphine-type alkaloids, 10 benzylisoquinoline-type alkaloids, 1 benzylphenethylamine-type alkaloid, 16 berberine-type alkaloids, 2 bisbenzylisoquinoline-type alkaloids, and 2 proaporphine-type alkaloids. Additionally, 6 anthraquinones, 5 flavonoids, 14 isoflavonoids, 3 phenanthrenequinones, 9 phenolic acids, 1 polyphenol, 4 saponins, and 8 triterpenes were also marked. 30 min after GQD was administered to mice by gavage, a total of 70 compounds were detected in the ileal contents, and after 240 min, 46 components were still detectable (Table 3). Some of them may have activity that stimulates the secretion of GLP-1 in the gut.

**Table 3:** Compounds annotated in GQD and the ileum contents

Peak NO	Compounds	Taxonomy	Formula	RT in GQD	Ileum content (min after gavage)				Possible source
					30	60	120	240	
1	Glucosyringic acid	Phenolic acids	C_15_H_20_O_10_	3.69	+	+	−	−	Dan shen
2	Salvianic acid A*	Phenolic acids	C_9_H_10_O_5_	4.15	+	+	−	−	Dan shen
3	Protocatechuic acid	Phenolic acids	C_7_H_6_O_4_	4.73	+	+	−	−	Dan shen
4	Protocatechuic acid 4-glucoside	Phenolic acids	C_13_H_16_O_9_	4.98	+	+	+	+	Dan shen
5	Puerarin-3'-hydroxy-4'-O-beta-D-glucopyranoside	Isoflavonoids	C_27_H_30_O_15_	5.47	+	+	+	+	Ge gen
6	Puerarin-4'-O-beta-D-glucopyranoside	Isoflavonoids	C_27_H_30_O_14_	5.73	+	+	+	+	Ge gen
7	Stepharine	Proaporphine alkaloids	C_18_H_19_NO_3_	5.81	−	−	−	−	He ye
8	Protocatechualdehyde*	Polyphenols	C_7_H_6_O_3_	6.36	+	+	−	−	Dan shen
9	Higenamine*	Benzylisoquinoline alkaloids	C_16_H_17_NO_3_	6.44	+	+	−	−	He ye
10	Daidzein-4',7-diglucoside	Isoflavonoids	C_27_H_30_O_14_	6.48	+	+	−	−	Ge gen
11	N-Methylhigenamine	Benzylisoquinoline alkaloids	C_17_H_19_NO_3_	6.64	+	+	−	−	He ye
12	3'-Hydroxypuerarin	Isoflavonoids	C_21_H_20_O_10_	7.02	+	+	+	+	Ge gen
13	Lotusine	Benzylisoquinoline alkaloids	C_19_H_24_NO_3_ +	7.21	+	+	+	+	He ye
14	10-Methoxy-5,8,13,13a-tetrahydro-6H-isoquino[3,2-a]isoquinoline-2,3,9-triol	Benzylisoquinoline alkaloids	C_18_H_19_NO_4_	7.70	+	+	+	+	He ye
15	Isolotusine	Benzylisoquinoline alkaloids	C_19_H_24_NO_3_ +	7.86	+	+	+	+	He ye
16	Caffeic acid*	Phenolic acids	C_9_H_8_O_4_	8.11	+	+	−	−	Dan shen
17	Puerarin*	Isoflavonoids	C_21_H_20_O_9_	8.31	+	+	+	+	Ge gen
18	Isococlaurine	Benzylisoquinoline alkaloids	C_17_H_20_NO_3_ +	8.67	+	+	−	−	He ye
19	3'-Methoxypuerarin	Isoflavonoids	C_22_H_22_O_10_	8.72	+	+	+	+	Ge gen
20	Norarmepavine	Benzylisoquinoline alkaloids	C_18_H_21_NO_3_	8.86	−	−	−	−	He ye
21	Pronuciferine	Proaporphine alkaloids	C_19_H_21_NO_3_	9.17	−	−	−	−	He ye
22	Magnoflorine	Aporphine alkaloids	C_20_H_24_NO_4_ +	9.32	+	+	+	+	Huang lian
23	Daidzin*	Isoflavonoids	C_21_H_20_O_9_	9.83	+	+	+	+	Ge gen
24	Aequaline	Berberine alkaloids	C_19_H_21_NO_4_	9.83	+	+	+	+	He ye
25	Glycitin	Isoflavonoids	C_22_H_22_O_10_	10.31	+	+	+	+	Ge gen
26	3′-Hydroxy puerarin 6″-O-xyloside	Isoflavonoids	C_26_H_28_O_14_	10.57	+	+	+	+	Ge gen
27	Lycoranine B	Benzylphenethylamine alkaloids	C_18_H_13_NO_4_	10.70	+	+	+	+	Huang lian
28	Liensinine*	Bisbenzylisoquinoline alkaloids	C_37_H_42_N_2_O_6_	10.78	+	+	+	+	He ye
29	Armepavine	Benzylisoquinoline alkaloids	C_19_H_23_NO_3_	10.78	+	+	−	−	He ye
30	N-norarmepavine	Benzylisoquinoline alkaloids	C_18_H_21_NO_3_	11.03	+	+	−	−	He ye
31	Neopuerarin A	Isoflavonoids	C_21_H_20_O_9_	11.05	+	+	+	+	Ge gen
32	Calycosin-7-glucoside*	Isoflavonoids	C_22_H_22_O_10_	11.17	+	+	+	+	Huang qi
33	Neopuerarin B	Isoflavonoids	C_21_H_20_O_9_	11.65	+	+	+	+	Ge gen
34	Coclaurine	Benzylisoquinoline alkaloids	C_17_H_19_NO_3_	11.84	+	+	−	−	He ye
35	Quercetin 3-O-B-D glucuronide	Flavonoids	C_21_H_18_O_13_	11.96	+	+	+	+	He ye
36	Menisperine	Aporphine alkaloids	C_21_H_26_NO_4_ +	12.05	+	+	+	+	Huang lian
37	Asimilobine	Aporphine alkaloids	C_17_H_17_NO_2_	12.45	−	−	−	−	He ye
38	Stecepharine	Berberine alkaloids	C_21_H_26_NO_5_ +	12.55	+	+	+	+	Huang lian
39	Tetradehydroscoulerine	Berberine alkaloids	C_19_H_16_NO_4_ +	12.73	+	+	+	+	Huang lian
40	O-Nornuciferine	Aporphine alkaloids	C_18_H_19_NO_2_	12.91	+	+	−	−	He ye
41	Demethyleneberberine	Berberine alkaloids	C_19_H_18_NO_4_ +	12.94	+	+	+	+	Huang lian
42	Neferine	Bisbenzylisoquinoline alkaloids	C_38_H_44_N_2_O_6_	13.06	+	+	−	+	He ye
43	13-Hydroxyberberine	Berberine alkaloids	C_20_H_18_NO_5_ +	13.21	+	+	+	+	Huang lian
44	Stephabine	Berberine alkaloids	C_21_H_22_NO_5_ +	13.33	+	+	+	+	Huang lian
45	Salvianolic acid D	Phenolic acids	C_13_H_22_O_15_	13.35	+	+	−	−	Dan shen
46	Columbamine	Berberine alkaloids	C_20_H_20_NO_4_ +	14.44	+	+	+	+	Huang lian
47	Rosmarinic acid*	Phenolic acids	C_18_H_16_O_8_	14.50	+	+	−	−	Dan shen
48	Epiberberine	Berberine alkaloids	C_20_H_18_NO_4_ +	14.59	+	+	+	+	Huang lian
49	Jatrorrhizine*	Berberine alkaloids	C_20_H_20_NO_4_ +	14.74	+	+	+	+	Huang lian
50	Coptisine*	Berberine alkaloids	C_19_H_14_NO_4_ +	14.91	+	+	+	+	Huang lian
51	Salvianolic acid B*	Phenolic acids	C_36_H_30_O_16_	15.85	+	+	−	−	Dan shen
52	13-Methylepiberberine	Berberine alkaloids	C_21_H_20_NO_4_ +	15.93	+	+	+	+	Huang lian
53	Berberrubine*	Berberine alkaloids	C_19_H_16_NO_4_ +	15.97	+	+	+	+	Huang lian
54	Daidzein*	Isoflavonoids	C_15_H_10_O_4_	16.08	+	+	+	+	Huang qi/Ge gen
55	Nuciferine*	Aporphine alkaloids	C_19_H_21_NO_2_	16.50	+	+	−	−	He ye
56	Palmatine*	Berberine alkaloids	C_21_H_22_NO_4_ +	16.50	+	+	+	+	Huang lian
57	Salvianolic acid A*	Phenolic acids	C_26_H_22_O_10_	16.83	+	−	−	−	Dan shen
58	Berberine*	Berberine alkaloids	C_20_H_18_NO_4_ +	16.85	+	+	+	+	Huang lian
59	Luteolin*	Flavonoids	C_15_H_10_O_6_	17.23	−	−	−	−	Huang qi/Dan shen/Ge gen/Mai ya/He ye
60	Quercetin*	Flavonoids	C_15_H_10_O_7_	17.38	−	−	−	−	Huang qi
61	Dehydrocorydaline	Berberine alkaloids	C_22_H_24_NO_4_ +	17.84	+	+	+	+	Huang lian
62	13-Methylberberine	Berberine alkaloids	C_21_H_20_NO_4_ +	18.37	+	+	+	+	Huang lian
63	Kaempferol*	Flavonoids	C_15_H_10_O_6_	19.27	−	−	−	−	Huang qi/Dan shen/Shan zha/Mai ya/He ye
64	Isorhamnetin*	Flavonoids	C_16_H_12_O_7_	19.37	−	−	−	−	Huang qi/Dan shen/Mai ya/He ye
65	Astragaloside IV*	Saponins	C_41_H_68_O_14_	19.59	+	+	+	+	Huang qi
66	16-Oxo-alisol A	Triterpenes	C_30_H_48_O_6_	19.96	+	+	−	−	Ze xie
67	Chrysophanol*	Anthraquinones	C_15_H_10_O_4_	20.15	−	−	−	−	Jue mingzi
68	Formononetin*	Isoflavonoids	C_16_H_12_O_4_	20.17	+	+	−	+	Huang qi/Dan shen/Ge gen/Mai ya/He ye
69	Astragaloside II*	Saponins	C_43_H_70_O_15_	20.19	+	+	+	+	Huang qi
70	Aurantio-obtusin*	Anthraquinones	C_17_H_14_O_7_	20.24	+	+	−	+	Jue mingzi
71	Soyasaponin I	Saponins	C_48_H_78_O_18_	20.47	+	+	+	+	Ge gen/Huang qi
72	Rhein*	Anthraquinones	C_15_H_8_O_6_	21.23	−	−	−	−	Jue mingzi
73	16-Oxo-11-anhydroalisol A	Triterpenes	C_30_H_46_O_5_	21.51	−	−	−	−	Ze xie
74	Astragaloside I*	Saponins	C_45_H_72_O_16_	21.60	+	+	−	−	Huang qi
75	Obtusin*	Anthraquinones	C_18_H_16_O_7_	21.76	+	+	−	+	Jue mingzi
76	Obtusifolin*	Anthraquinones	C_16_H_12_O_5_	22.07	+	+	−	−	Jue mingzi
77	23-Acetyl alisol C*	Triterpenes	C_32_H_48_O_6_	22.40	−	−	−	−	Ze xie
78	Emodin*	[22]	C_15_H_10_O_5_	22.73	+	+	+	+	Jue mingzi
79	Dihydrotanshinone I	Phenanthrenequinones	C_18_H_14_O_3_	22.74	+	+	−	−	Dan shen
80	Alisol B	Triterpenes	C_30_H_48_O_4_	23.21	−	−	−	−	Ze xie
81	Cycloastragenol*	Triterpenes	C_30_H_50_O_5_	23.21	+	+	−	−	Huang qi
82	Cryptotanshinone*	Phenanthrenequinones	C_19_H_20_O_3_	23.89	+	+	+	+	Dan shen
83	Maslinic acid*	Triterpenes	C_30_H_48_O_4_	24.83	+	+	−	−	Shan zha
84	Tanshinone IIA*	Phenanthrenequinones	C_19_H_18_O_3_	25.14	−	−	−	−	Dan shen
85	Ursolic acid*	Triterpenes	C_30_H_48_O_3_	25.98	−	−	−	−	Shan zha
86	Alisol B 23-acetate*	Triterpenes	C_32_H_50_O_5_	26.10	−	−	−	−	Ze xie

^*^Identified by reference standards

### Compounds from GQD promoted GLP-1 secretion in NCI-H716 cells

**Fig. 5:**
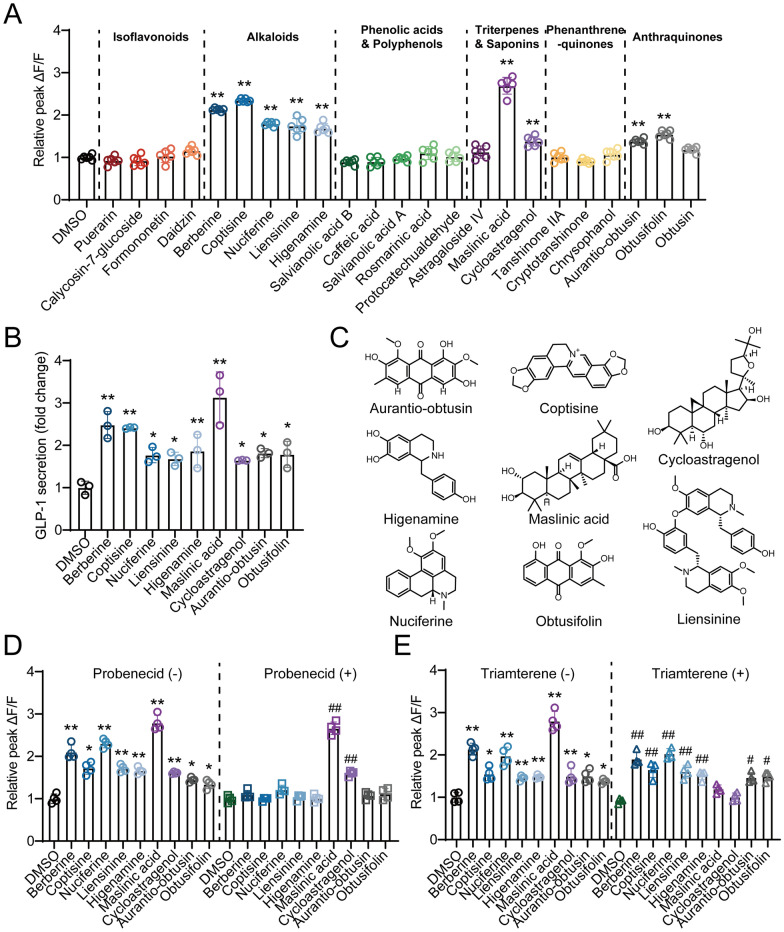
Compounds from GQD promoted GLP-1 secretion in NCI-H716 cells. **A** The effect of different compounds (25 μM) of GQD on the calcium influx in NCI-H716 cells (n = 6). **B** The effect of different compounds (25 μM) of GQD on the GLP-1 secretion in NCI-H716 cells (n = 3). **C** The chemical structures of the components in GQD that stimulate GLP-1 secretion. **D** The effect of 1 mM probenecid intervention on the promotion of calcium ion influx by GQD components (n = 4). **E** The effect of 5 μM probenecid intervention on the promotion of calcium ion influx by GQD components (n = 4). Compared with the DMSO group, #*p* < 0.05, ##*p* < 0.01. Compared with the DMSO group, *p < 0.05, **p < 0.01

Based on the distribution of GQD components in the ileum, the availability of commercial standards, and the representativeness of the components' structures, we selected 23 components from 6 major categories to investigate their effects on promoting calcium ion influx and GLP-1 release in NCI-H716 cells. As shown in Fig. 5A and B, berberine, coptisine, nuciferine, liensinine, higenamine, maslinic acid, cycloastragenol, aurantio-obtusin, and obtusifolin can increase intracellular calcium ion levels and promote GLP-1 secretion. Berberine and coptisine are berberine-type alkaloids from HL; Nuciferine, liensinine, and higenamine are alkaloids from HY, belonging to aporphine-type, bisbenzylisoquinoline-type, and benzylisoquinoline-type, respectively; Maslinic acid and cycloastragenol are triterpenes from SZ and HQ, respectively; Aurantio-obtusin and obtusifolin are anthraquinones from JM (Fig. 5C). As shown in Fig. 5D, probenecid can inhibit the calcium ion influx induced by berberine, coptisine, nuciferine, liensinine, higenamine, aurantio-obtusin, and obtusifolin, suggesting that these alkaloids and anthraquinones may activate bitter taste receptors on NCI-H716 cells. The effect of maslinic acid and cycloastragenol was abolished by triamterene (Fig. 5E), indicating that TGR5 may mediate the promoting effect of these two triterpenes on calcium ion influx.

### GQD remodeled the gut microbiota and its metabolism in HFD mice

**Fig. 6:**
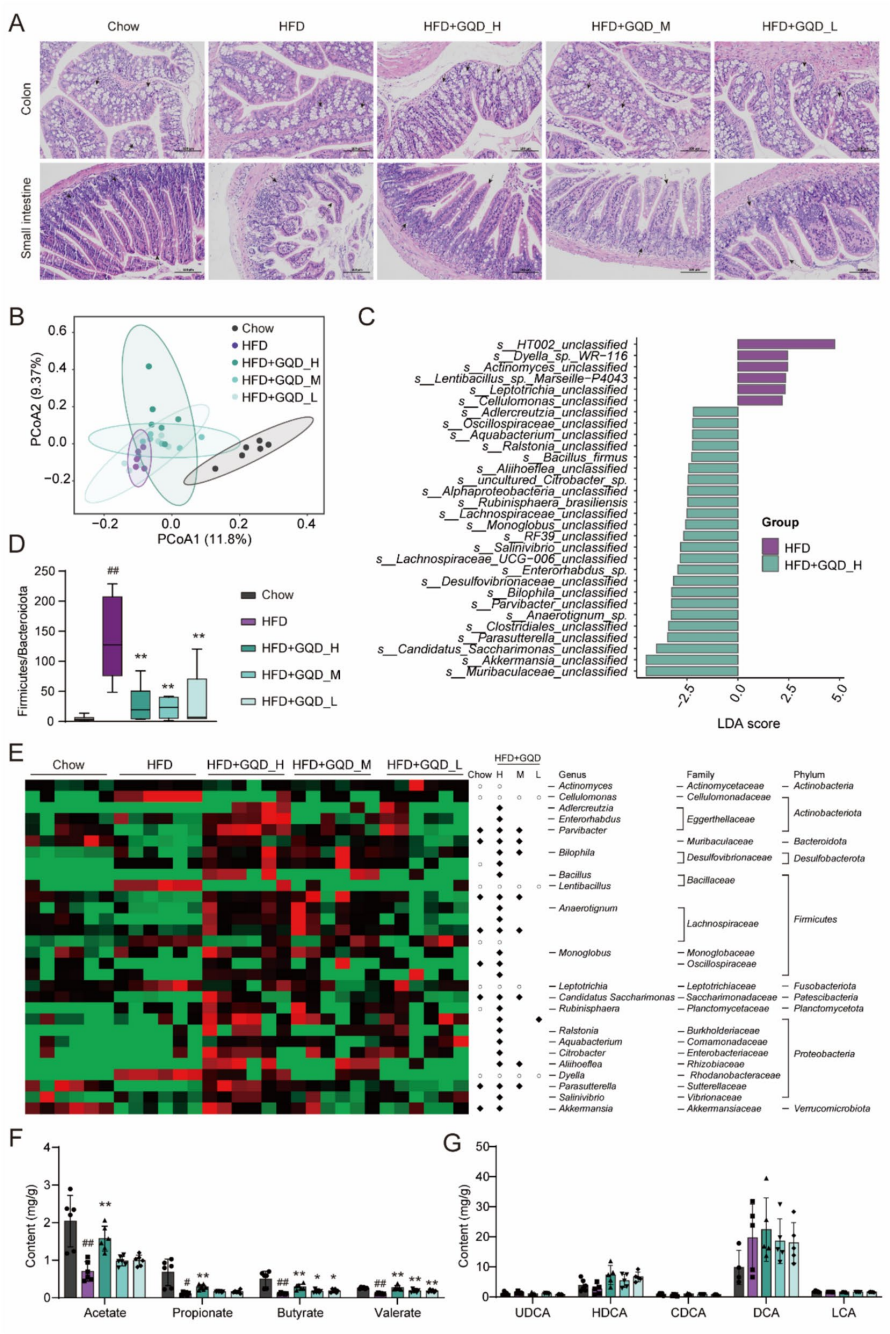
GQD remodeled the gut microbiota and its metabolism in HFD mice. **A** intestinal tissue sections stained with H&E (200 ×, scale bars: 100 µm). Solid arrows with solid lines represent crypts, solid arrows with dashed lines represent villi. **B** PCoA plot of colonic microbiota β-diversity. **C** Linear Discriminant Analysis (LDA) of species-level microbiota between the GQD_H and HFD groups. **D** Firmicutes to Bacteroidota ratio (n = 6). **E** Heatmap analysis of differential microbiota between the GQD_H and HFD groups across all groups. Hollow circles indicate significant downregulation compared to the HFD group, while solid diamonds indicate significant upregulation compared to the HFD group. **F** Colonic contents of SCFAs (n = 6). **G** Colonic contents of bile acids (n = 5). Compared with the Chow group, #*p* < 0.05, ##*p* < 0.01. Compared with the HFD group, *p < 0.05, **p < 0.01

In addition to directly stimulating GLP-1 secretion, GQD also upregulates *Gcg* gene expression in the colon of mice, increasing the reserve of GLP-1 precursor molecules and potentially raising GLP-1 levels in the long term. Studies have shown that the expression level of the colonic *Gcg* gene is closely related to the gut microbiota. Therefore, we first conducted a pathological analysis of the intestine. As shown in Fig. 6A, the crypts in the HFD mice became rounder and shorter, the villi were fragmented and shorter, and they were sparser. These changes were improved following treatment with GQD. Subsequently, we used 16S rDNA sequencing and metabolomics to examine the effects of GQD on the gut microbiota composition and metabolism in HFD mice. As shown in Fig. 6B, there was a significant difference in colonic microbiota composition between mice on a Chow and HFD diet, while GQD administration at different doses showed a trend of reversing gut dysbiosis. GQD_H, GQD_M, and GQD_L significantly reduced the Firmicutes-to-Bacteroidetes ratio in the colon (*p* < 0.01, Fig. 6D). To provide an insight into the changes in gut microbiota, LDA was performed to seek out the differential flora between the HFD group and GQD_H group (*p* < 0.05, |LDA|> 2.5, Fig. 6C). In HFD-fed mice, a total of 30 intestinal flora changed after GQD_H treatment. The abundance of *s_Muribaculaceae_unclassified* and *s_Akkermansia_unclassified* was significantly increased by administration of GQD_H. These 30 specific bacteria were further compared in all groups. Family analysis revealed that most of these altered intestinal flora belonging to *Lachnospiraceae* and *Eggerthellaceae*. Literature indicates that gut microbiota may upregulate *Gcg* expression through their metabolites, such as SCFAs and bile acids, by activating colonic SCFA receptors and TGR5, respectively. Metabolomic results showed that GQD_H, GQD_M, and GQD_L significantly increased butyrate and valerate levels in colonic contents (*p* < 0.01 or 0.05), and GQD_H also significantly elevated acetate and propionate levels (*p* < 0.01), as shown in Fig. 6E. However, the effect of GQD on colonic bile acid concentrations was minimal (Fig. 6F), with only GQD_H significantly increasing the level of HDCA in the colon (*p* < 0.05).

### The gut microbiota contributes to the anti-NAFLD effects of GQD

**Fig. 7:**
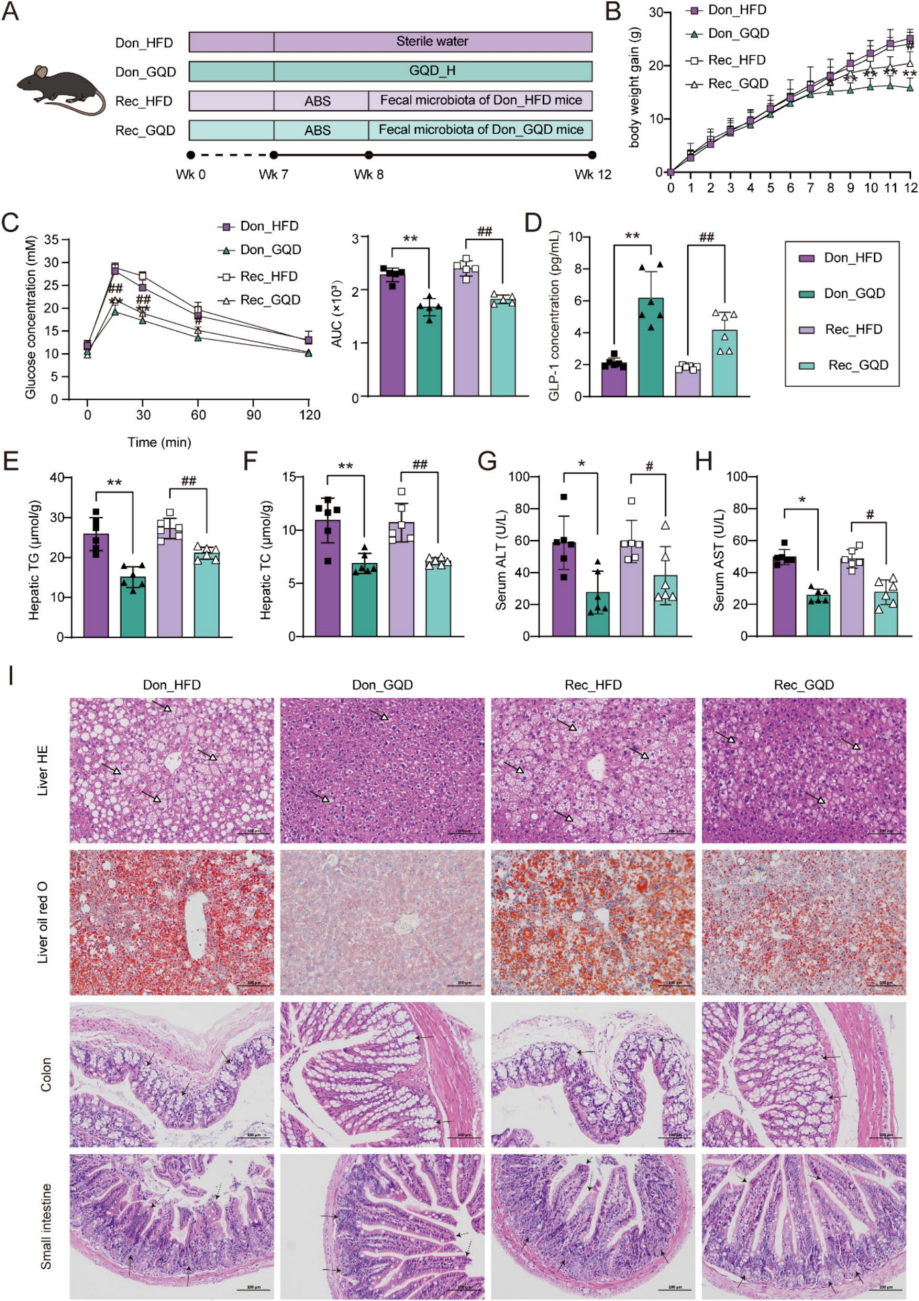
The gut microbiota contributes to the anti-NAFLD effects of GQD. **A** FMT experimental design. **B** Body weight gain (n = 6–8). **C** Blood glucose curves and AUC in OGTT (n = 5). **D** Serum GLP-1 levels (n = 6). **E** Hepatic TG contents (n = 6). **F** Hepatic TC contents (n = 6). **G** Serum ALT levels (n = 6). **H** Serum AST levels (n = 6). **I** Representative histological sections of liver, colon, and small intestine (200 ×, scale bars: 100 µm). Hollow arrows with solid lines represent lipid droplets, solid arrows with solid lines represent crypts, solid arrows with dashed lines represent villi. Compared with the Don_HFD group, *p < 0.05, **p < 0.01. Compared with the Rec_HFD group, #*p* < 0.05, ##*p* < 0.01

To investigate the role of the gut microbiota in the anti-NAFLD effects of GQD, we performed an FMT study. Fecal microbiota was collected from NAFLD model mice and GQD_H-treated mice, and transplanted via oral gavage into HFD-fed recipient mice (Fig. 7A). The results showed that recipient mice exhibited phenotypes similar to their respective donors. Compared with the Rec_HFD group, mice in the Rec_GQD group displayed attenuated body weight gain (*p* < 0.01, Fig. 7B), markedly improved insulin resistance (*p* < 0.01, Fig. 7C), significantly elevated GLP-1 levels (*p* < 0.01, Fig. 7D). More importantly, hepatic lipid accumulation was markedly reduced, accompanied by significant decreases in serum transaminase levels (*p* < 0.01 or 0.05, Fig. 7E–H). In addition, the crypt morphology and small intestinal villus structure of Rec_GQD mice were improved (Fig. 7I). Collectively, these findings suggest that the gut microbiota partially mediates the GQD-induced elevation of GLP-1 levels and the improvement of NAFLD-related phenotypes.

## Discussion

GLP-1 is an incretin hormone secreted by intestinal L cells, which are predominantly located in the distal small intestine and colon [17]. Once released into the bloodstream, GLP-1 exerts its effects by acting on the GLP-1 receptor (GLP1R) in the dorsomedial hypothalamus, thereby suppressing appetite [18]. Additionally, GLP-1 acts on pancreatic α and β cells, inhibiting glucagon secretion and promoting glucose-stimulated insulin secretion (GSIS) [19]. These mechanisms collectively reduce energy intake and alleviate hepatic steatosis associated with worsening insulin resistance, ultimately improving symptoms related to fatty liver disease [26, 27]. In this study, high and medium doses of GQD were observed to reduce energy intake in NAFLD mice and improve glucose tolerance, which may be attributed to elevated serum GLP-1 levels.

In NCI-H716 cells, GQD directly induced an elevation in intracellular calcium levels and stimulated the release of GLP-1. Various GPCRs expressed on the apical membrane of intestinal L cells, such as bitter taste receptors, sweet taste receptors, and TGR5, are known to respond to external stimuli and promote GLP-1 secretion [17]. It has been reported that berberine can activate the bitter taste receptor TAS2R38 in enteroendocrine cell lines such as STC-1 and NCI-H716, stimulating GLP-1 secretion in a phospholipase C-dependent manner [22, 28]. Alkaloids such as berberine contain a quaternary ammonium nitrogen atom. The resulting positive charge is likely the structural basis for their ability to activate bitter taste receptors and is also responsible for their hypolipidemic and hypoglycemic effects [29, 30]. The anthraquinone glycoside aloin is a ligand for the human bitter taste receptor T2R43, and in cells overexpressing human T2R43 (293 T cells), it promotes an increase in intracellular calcium levels [21]. Anthraquinone compounds may activate the bitter taste receptor T2R43 through key functional groups attached to their core structure, including hydroxyl and glucosyl moieties, thereby enabling them to regulate blood glucose levels [31, 32]. Isoflavonoids such as genistein and daidzein promote GLP-1 secretion in NCI-H716 cells, while the isoflavonoid glycoside glycitein does not have this effect [23]. These isoflavonoids have been identified as ligands for the human bitter taste receptors TAS2R14 or TAS2R39 [33]. A distinct difference between isoflavones and isoflavone glycosides may lie in the presence of a sugar moiety. Isoflavones, owing to their hydroxyl groups at specific positions, can bind to bitter taste receptors, resulting in various physiological functions [33]. Additionally, the triterpenoid compound maslinic acid has been identified as a receptor for TGR5 [24]. The polar functional groups in triterpenoids, such as hydroxyl, carboxyl, and carbonyl groups, may serve as the key structural basis for their specific binding to and activation of the TGR5 receptor [34, 35]. In our study, 70 components were identified from the ileum contents of mice gavaged with GQD, and the effects of 5 alkaloids, 4 anthraquinones, 4 isoflavonoid glycosides, 3 anthraquinones, and 5 phenolic compounds were examined. The results indicated that, in addition to berberine and coptisine, two berberine-type alkaloids, other alkaloids such as nuciferine, liensinine, and higenamine also promoted calcium ion influx and GLP-1 release in NCI-H716 cells. This effect was attenuated by the bitter taste receptor inhibitor probenecid, confirming that these alkaloids stimulate GLP-1 secretion via bitter taste receptors. Furthermore, it was confirmed that maslinic acid stimulates GLP-1 secretion in L cells via TGR5, similar to another triterpenoid compound, cycloastragenol. The 4 isoflavonoid glycosides tested did not show any regulatory effects on GLP-1 secretion.

In addition to exogenous substances, endogenous metabolites, particularly gut microbiota-derived secondary bile acids and SCFAs, are widely recognized as important regulators of GLP-1 [25, 36]. We observed that GQD intervention significantly elevated fecal SCFA levels in NAFLD mice, without impacting bile acid levels. It is well established that SCFAs are closely linked to alterations in gut microbiota composition. Specifically, an increased proportion of Firmicutes and Bacteroidetes has been identified as a key marker reflecting changes in the NAFLD-associated microbiota [37]. Comparison of gut microbial composition between HFD and GQD-treated HFD mice by 16SrDNA sequencing revealed that the ratio of Firmicutes-to-Bacteroidetes was significantly reduced after drug administration, implying that GQD helped to improve gut flora abundance and restore gut homeostasis. This alteration is associated with the synergistic effects of multiple active components in GQD, particularly alkaloids such as berberine and flavonoids such as baicalin, which have been reported to promote beneficial bacterial growth [38, 39]. At the species level, GQD treatment significantly enhanced the abundance of *Muribaculaceae* and *Akkermansia.* It is noteworthy that flavonoids and polysaccharides in GQD may be particularly beneficial for the proliferation of these microbial populations [40, 41]. Some studies have reported that *Akkermansia* could adhere to the mucus layer, participates in the production of SCFAs, especially acetate, propionate and butyrate, and regulates gene expression related to lipid metabolism and immune response [42–44]. *Muribaculaceae* are a family of bacteria within the order Bacteroidetes. *Muribaculaceae* produce short-chain fatty acids via endogenous (mucin glycans) and exogenous polysaccharides (dietary fibres) [45]. In addition, *Muribaculaceae* can regulate intestinal barrier function and the immune response, and they are considered a promising ‘next generation probiotic’ [46]. In addition to *Muribaculaceae* and *Akkermansia*, *Lachnospiraceae*, *Anaerotignum*, *Clostridiales* all showed a significant increase in abundance after administration of GQD. The *Lachnospiraceae* group, representing kind of butyrate-producing bacteria, has been found to maintain gut barrier integrity in mice and is negatively correlated with intestinal permeability [47]. *Anaerotignum* is one of the main intestinal bacteria that produce butyric and propionate which is beneficial for intestinal health [48]. *Clostridiales* produced mainly butyrate for intestinal modulation [49]. It is hypothesized that flavonoids, isoflavonoids, and triterpenoids in GQD, such as berberine, puerarin, and maslinic acid, may exert their effects by improving the gut microbiota, specifically by increasing the abundance of Akkermansia and Lachnospiraceae, thereby stimulating butyrate production [50–53]. Through its multi-component synergistic action, GQD fosters an intestinal environment conducive to the growth of these SCFA-producing bacteria, thereby indirectly facilitating GLP-1 secretion.

SCFAs produced by various human gut microbes serve as an energy source for the colonic epithelium and are sensed by host signaling pathways that modulate appetite and inflammation, as previously reported [54]. Numerous studies have demonstrated that SCFAs regulate metabolic disorders and immunity mainly through the inhibition of histone deacetylases and the activation of G protein-coupled receptors such as GPR41, GPR43 and GPR109a [55]. On the other hand, SCFAs like acetate and butyrate have been shown to improve glucose homeostasis by inducing gut production of glucagon-like peptide-1 (GLP-1), which in turn stimulate insulin secretion [54].GLP-1 is produced both in gut endocrine cells and in the brain, where it regulates islet function, satiety, and gut motility through hormonal and neural pathways [56]. Multiple reports have shown that SCFAs can stimulate GLP-1 secretion via GRP43 and GPR41, and can also increase serum GLP-1 levels and *Gcg* mRNA expression [43, 57]. Additionally, several herbs in GQD, such as Huanglian, Gegen have been been reported to modulate the intestinal flora and influence SCFA levels. Specifically, polysaccharides and berberine from Huanglian have been have been shown to increase the abundance of *Akkermansia* and *Bacteroides* which had strong positive correlations with the contents of SCFAs [58]. Puerarin in Gegen also could affect the abundance of *Peptococcaceae* and *Closteridiales*, leading to changes in SCFAs [59].

Notably, although our findings support a role for TAS2R38 and TGR5 in mediating GQD-induced GLP-1 secretion, these conclusions are based on pharmacological inhibition rather than genetic loss-of-function models. In our study, both the bitter receptor inhibitor probenecid and the TGR5 inhibitor triamterene attenuated the acute GLP-1–stimulating effect of GQD in vivo, suggesting that activation of these receptors contributes to the observed response. However, pharmacological inhibitors may not fully recapitulate receptor-specific deletion, and off-target effects cannot be completely excluded. Therefore, definitive confirmation of receptor dependency will require future studies using intestinal L-cell–specific or global knockout models of bitter receptor and *Gpbar1*. Such genetic approaches will be essential to precisely define the contribution of each receptor to the metabolic actions of GQD.

## Conclusion

**Fig. 8:**
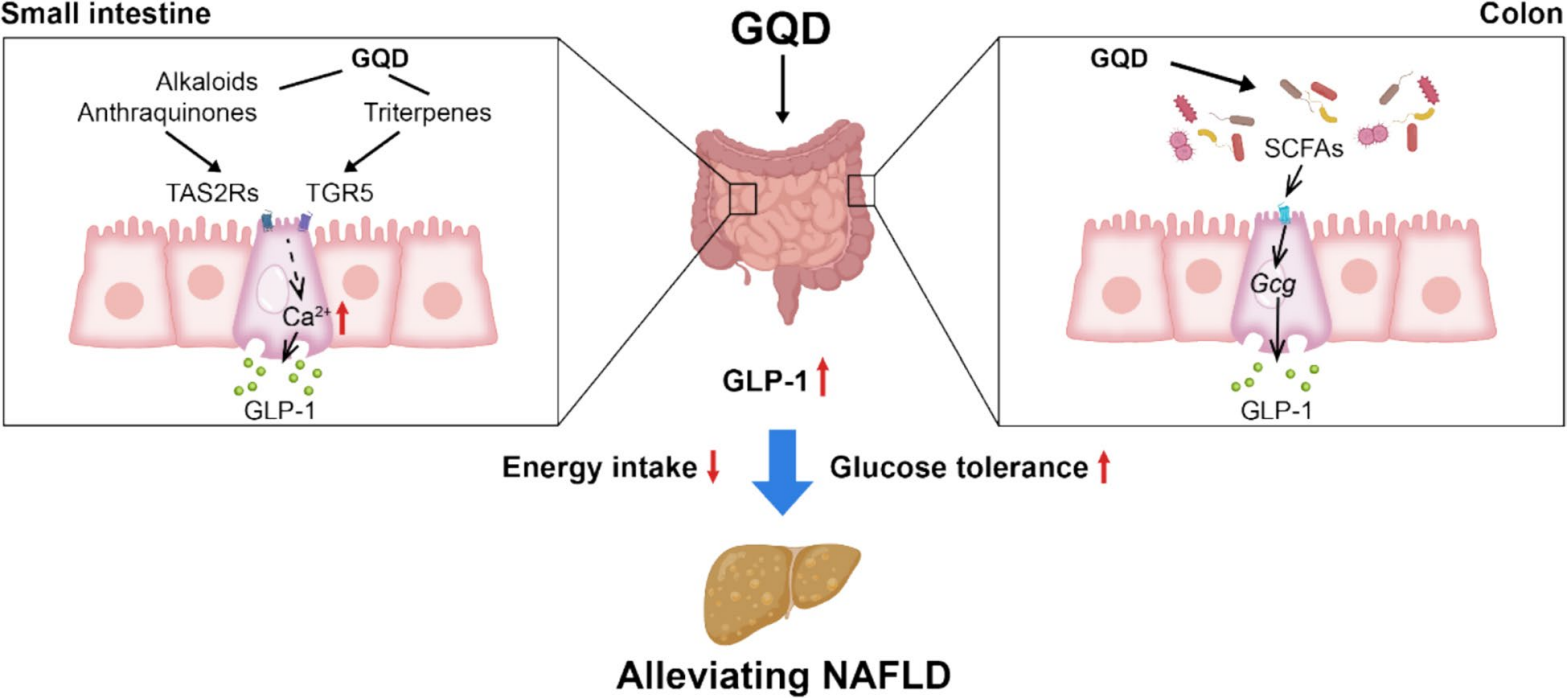
Multiple mechanisms by which GQD elevates GLP-1 levels contribute to the improvement of NAFLD. Alkaloids, triterpenes, and anthraquinones of GQD activate TAS2Rs and TGR5 in the small intestine, leading to increased intracellular calcium influx and subsequent stimulation of GLP-1 secretion. Meanwhile, GQDs modulate the intestinal flora gold should affect the content of SCFAs, which in turn enhance the colonic *Gcg* expression, also further increasing GLP-1 levels. The increased GLP-1 improves insulin sensitivity, and separately, its satiety-promoting effect reduces energy intake. Together, these actions contribute to the amelioration of NAFLD

In summary, our study showed that GQD can activate GPCRs, including bitter taste receptors and TGR5, in intestinal endocrine cells, promoting GLP-1 secretion. Simultaneously, GQD regulates gut microbiota composition and metabolism, increasing SCFA levels and *Gcg* gene expression, leading to sustained elevation of GLP-1 levels (Fig. 8). This result provides a new drug regimen for the treatment of NAFLD.

## Supplementary Information

Supplementary Material 1.

## Data Availability

No datasets were generated or analysed during the current study.

## Abbreviations

NAFLD: Non-alcoholic fatty liver disease
GQD: Ge-Lian Qi-Shen Decoction
HFD: High-fat diet
GLP-1: glucagon-like peptide-1
GPCRs: G-protein-coupled receptors
TGR5: Takeda G-protein-coupled receptor 5
SCFAs: Short-chain fatty acids
IR: Insulin resistance
HCC: Hepatocellular carcinoma
JZMA: Jiang Zhi Mai An
HQ: Huang qi (the root of *Astragalus membranaceus* (Fisch.) Bunge var. mongholicus (Bunge) Hsiao)
DS: Dan shen (the root and rhizome of *Salvia miltiorrhiza* Bunge)
GG: Ge gen (the root of *Pueraria lobata* (Willd.) Ohwi)
HL: Huang lian (the rhizome of *Coptis chinensis* Franch.)
ZX: Ze xie (the rhizome of Alisma orientale (Sam.) Juzep.)
JM: Jue mingzi (the seed of *Cassia obtusifolia* L.)
SZ: Shan zha (the fruit of *Crataegus pinnatifida* Bunge)
MY: Mai ya (the germinated fruit of *Hordeum vulgare* L.)
HY: He ye (the leaf of *Nelumbo nucifera* Gaertn.)
OGTT: Oral glucose tolerance test
TG: Triglycerides
TC: Total cholesterol
HDL-C: High-density lipoprotein cholesterol
LDL-C: Low-density lipoprotein cholesterol
ALT: Alanine aminotransferase
AST: Aspartate aminotransferase
KRH: Krebs-Ringer HEPES
RFU: Relative fluorescence units
siRNA: Small interfering RNA
3NPH: 3-nitrophenylhydrazine
EDC: N-(3-dimethylaminopropyl)-N’-ethylcarbodiimide
LCA: Lithocholic acid
HDCA: Hyodeoxycholic acid
UDCA: Ursodeoxycholic acid
CDCA: Chenodeoxycholic acid
DCA: Deoxycholic acid
FMT: Fecal microbiota transplantation
AUC: Area under the curve
PTC: Phenylthiocarbamide
LDA: Linear discriminant analysis
GLP1R: GLP-1 receptor
GSIS: Glucose-stimulated insulin secretion
