# Supplementary material for: Targeting G-protein-coupled receptors and gut microbiota: Ge-Lian Qi-Shen decoction elevates GLP-1 to combat non-alcoholic fatty liver disease
Source: Chin Med. 2026 Jan 24;21:51. doi: 10.1186/s13020-025-01305-9 (PMC12831259; doi:10.1186/s13020-025-01305-9)
Supplement: Supplementary file 1 — Supplementary Material 1. [file 13020_2025_1305_MOESM1_ESM.docx]

**Targeting G-Protein-Coupled Receptors and Gut Microbiota: Ge-Lian Qi-Shen Decoction Elevates GLP-1 to Combat Non-Alcoholic Fatty Liver Disease**

Supplementary information:

Table S1 qPCR Primer Sequences.

| Gene | Species | Forward (5’-3’) | Reverse (5’-3’) |
| --- | --- | --- | --- |
| *Gcg* | Mouse | AAGAGGAACCGGAACAACATTG | GCCCTCCAAGTAAGAACTCACA |
| *Rn18s* | Mouse | GGCCGATGACGAGCCC | TGTCTTTGGAACTTTGTCTGCA |
| *GPBAR1* | Human | CCTGGCAAGCCTCATCATCA | CCAGCAGTAGGCTCAGGAAG |
| *TAS2R38* | Human | GACTGCTGTTCCTGAGTGCT | CAGAGGTTGGCTTGGTTTGC |
| *ACTB* | Human | GCCGAGGACTTTGATTGC | CCTGTGTGGACTTGGGAGA |

Table S2 siRNA Oligonucleotide Target Sequences.

| Gene (Transcript accession) | Oligo name | Target (5’-3’) |
| --- | --- | --- |
| GPBAR1 (NM_170699.3) | *GPBAR1-*1 | Sense strand: GGUCCUGCCUCCUCGUCUATT |
|  |  | Antisense strand: UAGACGAGGAGGCAGGACCTT |
|  | *GPBAR1-*2 | Sense strand: GCCUCAUCAUCACCGCGAATT |
|  |  | Antisense strand: UUCGCGGUGAUGAUGAGGCTT |
|  | *GPBAR1-*3 | Sense strand: GGCUGGGCGAUCAGCGCUATT |
|  |  | Antisense strand: UAGCGCUGAUCGCCCAGCCTT |
| TAS2R38 (NM_176817.5) | *TAS2R38-*1 | Sense strand: GGCAGAUUAAAGAUCUCAATT |
|  |  | Antisense strand: UUGAGAUCUUUAAUCUGCCTT |
|  | *TAS2R38-*2 | Sense strand: GGCACAUGAGGACAAUGAATT |
|  |  | Antisense strand: UUCAUUGUCCUCAUGUGCCTT |
|  | *TAS2R38-*3 | Sense strand: GGGAUGUAGUGAAGAGGCATT |
|  |  | Antisense strand: UGCCUCUUCACUACAUCCCTT |


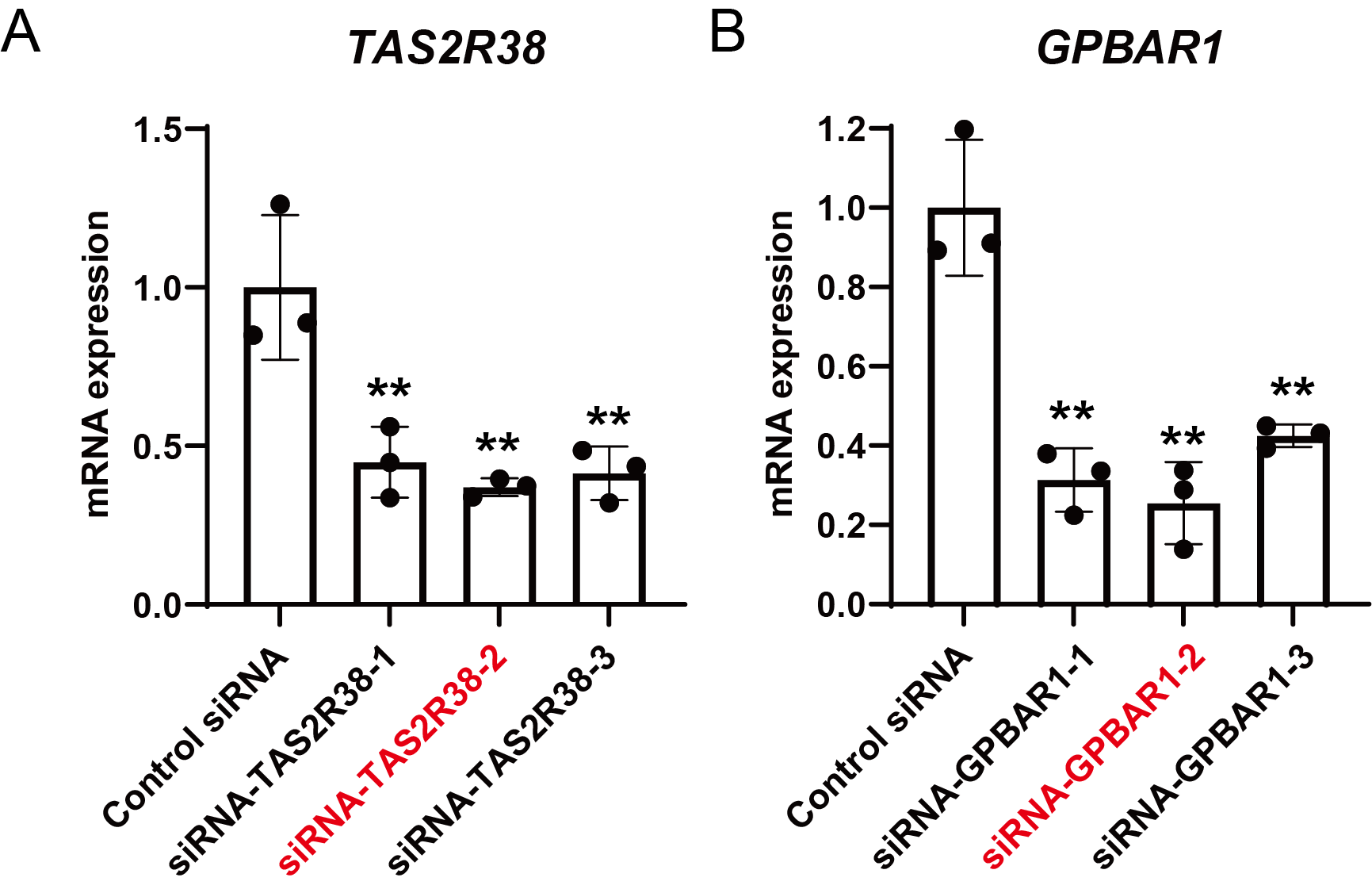


Fig. S1 siRNA knockdown efficiency for the *GPBAR1* gene (**A**) and the *TAS2R38* gene (**B**). The siRNA oligonucleotides (target sequences listed in Table S2) highlighted in red were selected for downstream experiments.


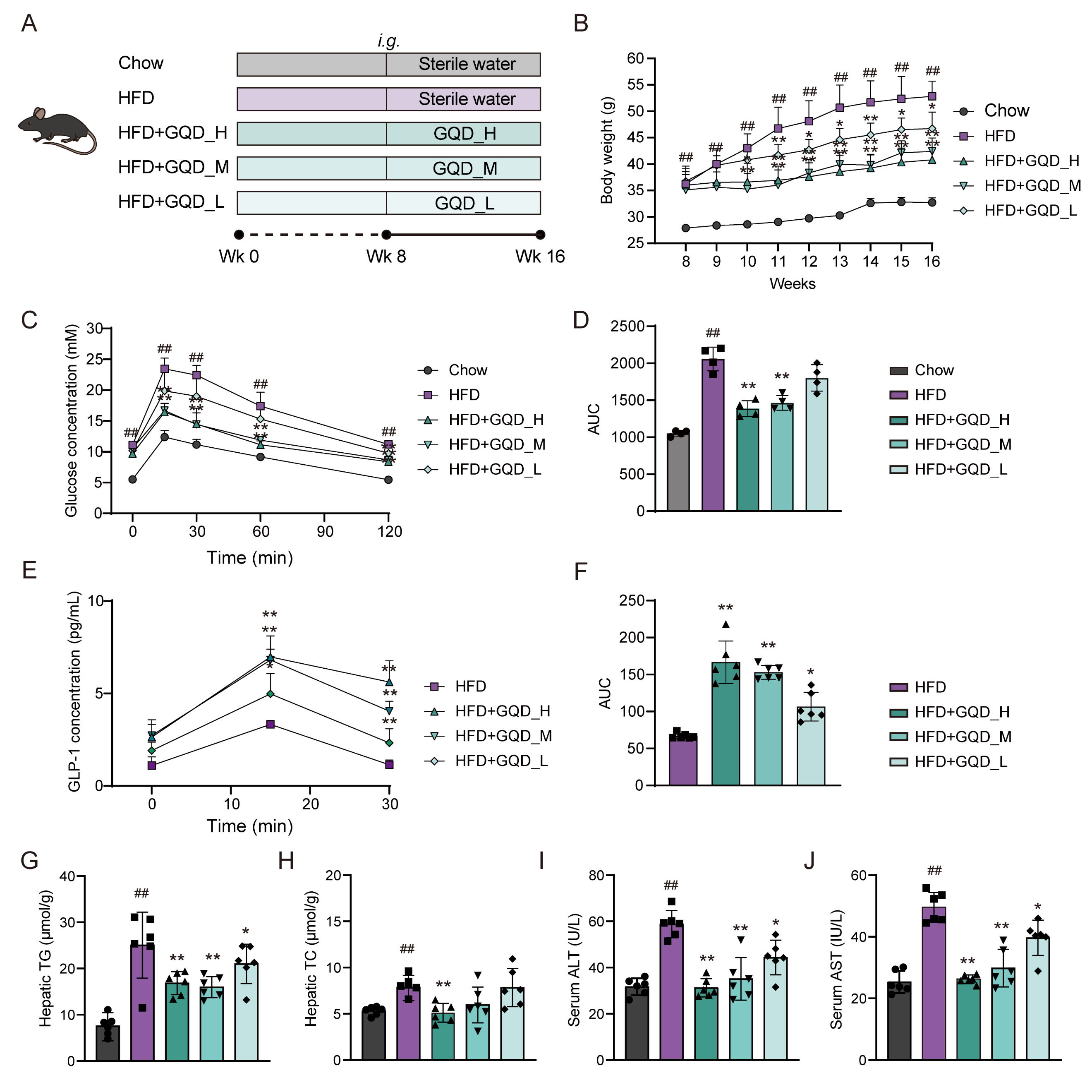
Fig. S2 Long-term GQD administration promoted GLP-1 secretion in HFD-fed mice. **A** Animal experimental design. **B** Body weight (n=6). **C** Blood glucose curves in OGTT (n=4). **D** The area under the curve (AUC) of blood glucose curves in OGTT (n=4). **E** Serum GLP-1 concentration (n=6). **F** The area under the curve (AUC) of serum GLP-1 curves. **G** Hepatic TG contents (n=6). **H** Hepatic TC contents (n=6). **I** Serum ALT levels (n=6). **J** Serum AST levels (n=6). Compared with the Chow group, ## p<0.01. Compared with the HFD group, * p<0.05, ** p<0.01.


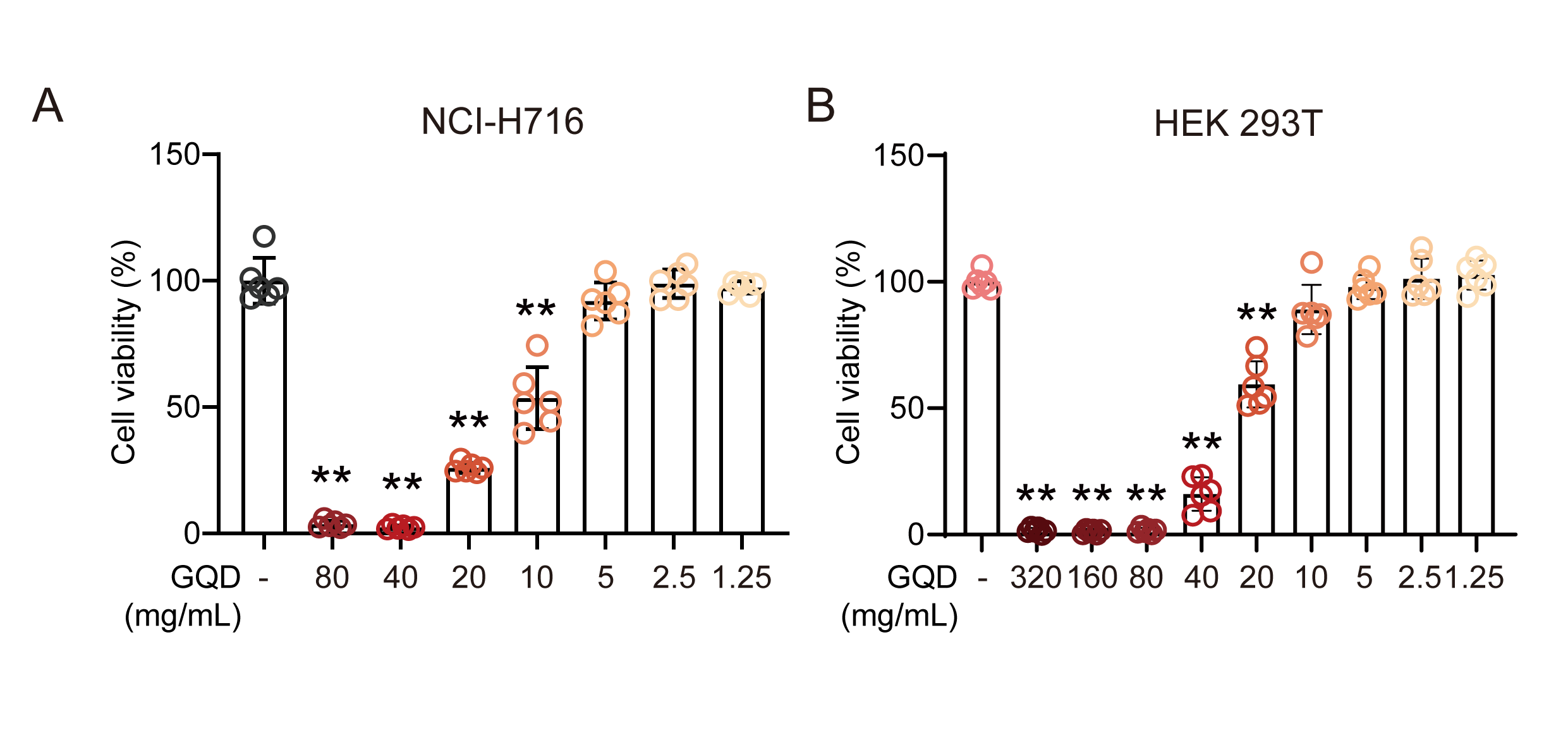


Fig. S3 **A** CCK-8 assay of GQD in NCI-H716 cells. **B** CCK-8 assay in HEK 293T cells.
